# Association between psychiatric disorders and glioma risk: evidence from Mendelian randomization analysis

**DOI:** 10.1186/s12885-024-11865-y

**Published:** 2024-01-23

**Authors:** Wenzhuo Yang, Yu Han, Changjia He, Sheng Zhong, Fei Ren, Zhongping Chen, Yonggao Mou, Ke Sai

**Affiliations:** 1 Department of Neurosurgery/Neuro-oncology, Sun Yat-sen University Cancer Center, 510060 Guangzhou, China; 2 State Key Laboratory of Oncology in South China, Collaborative Innovation Center for Cancer Medicine, Sun Yat-sen University Cancer Center, 510060 Guangzhou, P. R. China; 3 The Second Bethune Clinical Medical College, Jilin University, 130041 Changchun, P. R. China

**Keywords:** Mendelian randomization, Schizophrenia, Glioma, Etiology

## Abstract

**Background:**

Observational studies have explored the association of psychiatric disorders and the risk of brain cancers. However, the causal effect of specific mental illness on glioma remains elusive due to the lack of solid evidence.

**Methods:**

We performed a two-sample bidirectional Mendelian randomization (MR) analysis to explore the causal relationships between 5 common psychiatric disorders (schizophrenia, major depressive disorder, bipolar disorder, autism spectrum disorder, and panic disorder) and glioma. Summary statistics for psychiatric disorders and glioma were extracted from Psychiatric Genomics Consortium (PGC) and 8 genome-wide association study (GWAS) datasets respectively. We calculated the MR estimates for odds ratio of glioma associated with each psychiatric disorder by using inverse-variance weighting (IVW) method. Sensitivity analyses such as weighted median estimator, MR-Egger and MR-PRESSO were leveraged to assess the strength of causal inference.

**Results:**

A total of 30,657 participants of European ancestry were included in this study. After correction for multiple testing, we found that genetically predicted schizophrenia was associated with a statistically significant increase in odds of non-glioblastoma multiforme (non-GBM) (OR = 1.13, 95% CI: 1.03–1.23, *P* = 0.0096). There is little evidence for the causal relationships between the other 4 psychiatric disorders with the risk of glioma.

**Conclusions:**

In this MR analysis, we revealed an increased risk of non-GBM glioma in individuals with schizophrenia, which gives an insight into the etiology of glioma.

**Supplementary Information:**

The online version contains supplementary material available at 10.1186/s12885-024-11865-y.

## Introduction

Glioma is a frequent tumor in the central nervous system (CNS), representing approximately 25% of primary brain tumors and 80% of intracranial malignancies in adults [1]. The nature of aggressive growth and inherent resistance to conventional therapies make glioma one of the most lethal diseases. The median overall survival (OS) is only less than 2 years for the most common subtype glioblastoma multiforme (GBM) [2]. Over the last four decades, considerable endeavors have been undertaken to ascertain the etiological factors contributing to gliomagenesis. Noteworthy risk factors, such as radiation exposure and genetic alterations have been linked to susceptibility to glioma [3]. However, the etiology of gliomas has not been fully elucidated.

Previous epidemiological studies have suggested potential associations between psychiatric disorders and the susceptibility to brain cancers. Grinsphpoon et al. showed that men with schizophrenia were less likely to develop brain cancers [4]. Dalton et al. reported that dysthymia increased the risk of brain cancers, with a standardized incidence ratio (SIR) of 1.18 (95% CI: 1.13–1.23) [5]. A regional population-based study yielded no evidence of an association between bipolar disorder and the incidence of brain cancers [6]. However, conventional observational studies have been constrained by the poorly-defined histological classification of brain cancers and potential methodological biases, leading to inconclusive or conflicting conclusions regarding the causal connections between psychiatric disorders and glioma.

Mendelian randomization (MR) is a powerful epidemiological approach that uses genetic variants robustly associated with exposure to estimate the causal effect on outcome. MR studies are less susceptible to reverse causality and unmeasured confounding due to the fixed nature of genetic variants at conception. Accordingly, MR analyses have been widely employed to investigate the causal relationship between risk factors and diseases.

To shed light on the causal effect of mental illness on glioma, we applied a two-sample bidirectional MR analysis and explored the associations of single nucleotide polymorphisms (SNPs) related to 5 frequent psychiatric disorders and glioma risk by using summary data from Psychiatric Genomics Consortium (PGC) and 8 glioma GWAS datasets.

## Methods

A two-sample bidirectional MR analysis was performed by utilizing GWAS data. Approval of ethics was not required for this study because all data were from publicly available GWAS publications and no individual-level data were used.

### GWAS data resource for psychiatric disorders and glioma

Five psychiatric disorders including schizophrenia (SCZ), major depressive disorder (MDD), bipolar disorder (BD), autism spectrum disorder (ASD), and panic disorder (PD), were investigated. SNPs associated with each of the psychiatric disorders were obtained from GWAS statistics provided by Psychiatric Genomics Consortium (PGC) (https://pgc.unc.edu/). In this study, 130,644 participants for SCZ (53,386 cases and 77,258 controls), 500,199 for MDD (170,756 cases and 329,443 controls), 413,466 for BD (41,917 cases and 371,549 controls), 46,350 for ASD (18,381 cases and 27,969 controls) and 10,240 for PD (2,248 cases and 7,992 controls), were included. All the participants were European descendants.

Summary data of genetic susceptibility to glioma were retrieved from 8 GWAS datasets, in which more than 10 million SNPs were imputed and related to 12,488 glioma cases and 18,169 controls of European ancestry (Supplementary Table 1). A dichotomous histological stratification of all gliomas, namely, GBM (6,183 cases) and non-GBM (5,820 cases), was used [7].

### Mendelian randomization

#### Selection of instrumental variables

We estimated the causal effects of psychiatric disorders on glioma by using genetic variants as instrumental variables (IVs) for exposure. An IV is required to be associated with exposure, but not with unmeasured confounding factors for causality. Additionally, a valid IV is independent of any causal pathway to outcome other than through exposure [8]. For each of the psychiatric disorders, SNPs with a genome-wide significance of *P* < 5 × 10^− 8^ were obtained from PGC. We used linkage disequilibrium clumping approach to identify independent SNPs with a stringent threshold of *r*^*2*^ < 0.01 [9]. The F-statistic for each single nucleotide polymorphism (SNP) was estimated by squaring the SNP’s effect on exposure and dividing it by the variance of the SNP’s effect on exposure, employing the approximation proposed by Bowden et al [10]. Subsequently, the mean F-statistic for the exposure was calculated. SNPs with an F-statistic less than 10 were excluded [11].

### Statistical analysis

In the two-sample MR analysis, we leveraged a random-effects inverse variance-weighted (IVW) approach that combines the genetic association estimates across multiple variants to assess the causal relationship between psychiatric disorders and glioma [9]. IVW method efficiently provides precise causal effect estimate but can be biased when pleiotropic variants are present. Accordingly, three sensitivity analyses under different model assumptions of pleiotropy, namely weighted median estimator (WME), MR-Egger regression and MR pleiotropy residual sum and outlier (MR-PRESSO) method, were applied to address the strength of the primary causal inference. WME assumes that at least half of genetic instruments are valid and calculates the causal effect estimate by using the median of the IV weights. WME is consistent for the true causal effect even when up to 50% of invalid IVs are present [12]. MR-Egger regression is a modified IVW approach, which initially estimates the pleiotropy parameter by adding an intercept term and subsequently uses it to adjust for the causal effect. A non-zero intercept implicates the existence of unbalanced pleiotropy. Regardless of the superior robustness to pleiotropy, MR-Egger regression is less powerful to detect a small causal effect compared to WME and IVW method [13]. MR-PRESSO, on the other hand, is used to identify and correct the horizontal pleiotropic effect by the removal of potential outlier IVs [14]. In additon, Mendelian randomization based on constrained maximum likelihood (MR-cML) was employed to control correlated and uncorrelated pleiotropic effects [15]. We investigated the heterogeneity among variant-specific causal estimates with Cochran’s Q statistic and applied leave-one-out analysis to examine whether the estimate of the causal effect is dominated by a particular genetic variant [16, 17].

Results were presented in term of odds ratios (OR) with 95% confidence intervals (CI) per logOR unit change for each psychiatric disorder. We applied Bonferroni correction to adjust for multiple comparison. We considered a *P* < 0.05 as evidence for a suggestive causal association, and a *P* < 0.01 (i.e. 0.05/5 psychiatric disorders) as a significant association. Statistical analyses were counted by using R version 4.1.2.

## Results

### Identification of genetic instruments

The number of SNPs utilized for each psychiatric disorder and glioma risk ranged from 8 to 128. All the SNPs had a minimum F-statistic value greater than 10, indicating the absence of weak instrumental variants (Supplementary Tables 2–7).

### Causal effect of psychiatric disorders on gliomas

In the primary MR analysis, we explored the causal associations between each of 5 psychiatric disorders and glioma by performing the IVW approach under a random-effects model. Suggestive evidence of causal effect of SCZ on all-glioma (OR = 1.07, 95% CI = 1.01–1.14, *P* = 0.0260) was identified. However, the causal association was dominantly influenced by the particular SNP rs13195636 at 6p22.3, which has an unknown biological function. After removal of rs13195636, the suggestive causal effect became non-significant (OR = 1.06, 95% CI = 1.00-1.13, *P* = 0.066). Lack of association between other 4 mental illness and risk of all-glioma was found.

**Fig. 1:**
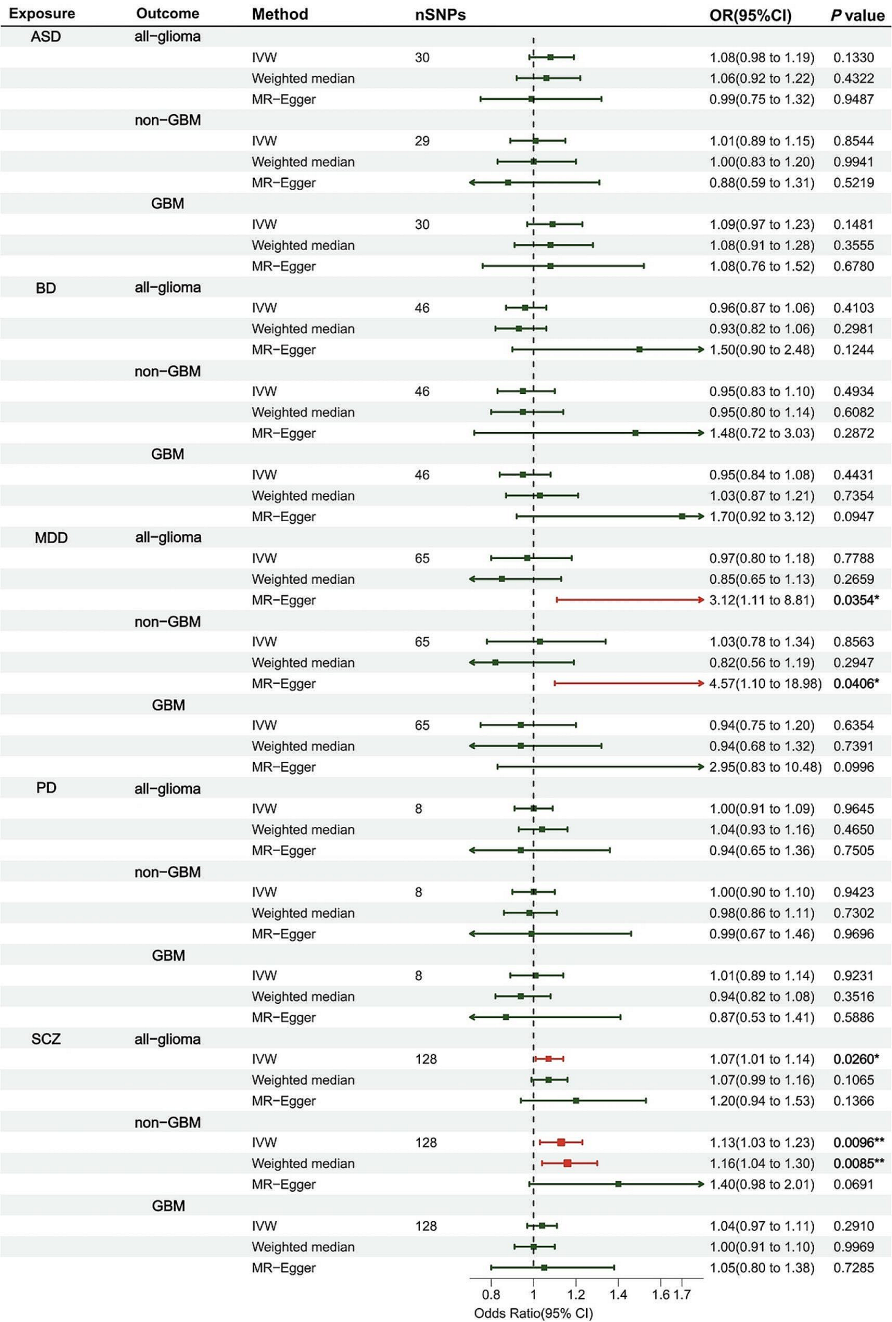
Forrest plot of multivariable MR estimates for the causal associations between psychiatric disorders and glioma Results are represented as odds ratios (OR) and 95% CIs per logOR unit change in the exposure. *: suggestive association (0.01 < *P* < 0.05); **: significant association (*P* < 0.01)

Considering the possibility that the risk of glioma might be associated with psychiatric disorders in a subtype-specific manner, we investigated the causal effects in GBM and non-GBM separately. We found a positive association between SCZ and the risk of non-GBM (OR = 1.13, 95% CI = 1.03–1.23, *P* = 0.0096). The consistency in the estimate was also demonstrated by WME and MR-cML. MR-Egger intercept indicated negligible horizontal pleiotropy. Additionally, we applied MR-PRESSO global test to detect SNP outliers and corrected with MR-PRESSO outlier test and found similar estimate (OR = 1.14, 95% CI:1.045–1.247, *P* = 0.0038, *P* for MR-PRESSO distortion test = 0.804). Moreover, the leave-one-out analysis indicated that the causal estimate was not reliant on any single SNP. Except for the association between SCZ and the risk of non-GBM, there is little evidence for the causal relationships between all 5 psychiatric disorders and either GBM nor non-GBM. The results of causal estimates and sensitivity analyses were provided in Fig. 1; Table 1, Supplementary Tables 8 and Supplementary Figs. 1–15.

**Table 1:** Sensitivity analyses including weighted median estimator, MR-Egger regression, Cochran’s Q statistic and MR-Egger intercept test for the causal association between psychiatric disorders and glioma

			Weighted median estimator				MR-Egger				Heterogeneity (*P*)	Pleiotropy (*P*)
Exposure	Outcome	SNPs	*P*	OR	Lower 95%CI	Upper 95%CI	*P*	OR	Lower 95%CI	Upper 95%CI		
PD	all-glioma	8	0.465	1.042	0.935	1.160	0.750	0.940	0.651	1.357	0.208	0.750
ASD		30	0.432	1.059	0.918	1.222	0.949	0.991	0.746	1.316	0.527	0.541
SCZ		128	0.106	1.071	0.985	1.165	0.137	1.203	0.945	1.533	0.054	0.333
BD		46	0.298	0.933	0.820	1.063	0.124	1.497	0.904	2.478	0.133	0.084
MDD		65	0.266	0.854	0.648	1.127	0.035^*^	3.121	1.106	8.807	0.776	0.029^*^
PD	GBM	8	0.347	0.936	0.816	1.073	0.589	0.868	0.533	1.412	0.105	0.560
ASD		30	0.356	1.083	0.915	1.281	0.678	1.077	0.762	1.523	0.665	0.933
SCZ		128	0.997	1.000	0.906	1.103	0.728	1.050	0.799	1.380	0.396	0.931
BD		46	0.735	1.029	0.873	1.213	0.095	1.699	0.924	3.123	0.149	0.064
MDD		65	0.739	0.945	0.676	1.320	0.0996	2.948	0.829	10.48	0.854	0.078
PD	non-GBM	8	0.730	0.977	0.854	1.114	0.969	0.992	0.674	1.459	0.625	0.983
ASD		29	0.994	0.999	0.833	1.198	0.522	0.876	0.587	1.307	0.528	0.460
SCZ		128	0.009^**^	1.160	1.039	1.296	0.069	1.402	0.977	2.013	0.000^**^	0.223
BD		46	0.608	0.954	0.795	1.144	0.287	1.482	0.724	3.031	0.043^*^	0.223
MDD		65	0.295	0.817	0.561	1.192	0.041^*^	4.567	1.099	18.976	0.203	0.040^*^

PD = panic disorder, ASD = autism spectrum disorder, SCZ = schizophrenia, BD = bipolar disorder, MDD = major depressive disorder

* Showed significant differences *P* < 0.05, ** Bonferroni corrected *P* < 0.01

Heterogeneity was examined by using Cochran’s Q statistic

Pleiotropy was assessed with MR-Egger intercept test

### Causal effect of gliomas on psychiatric disorders

**Fig. 2:**
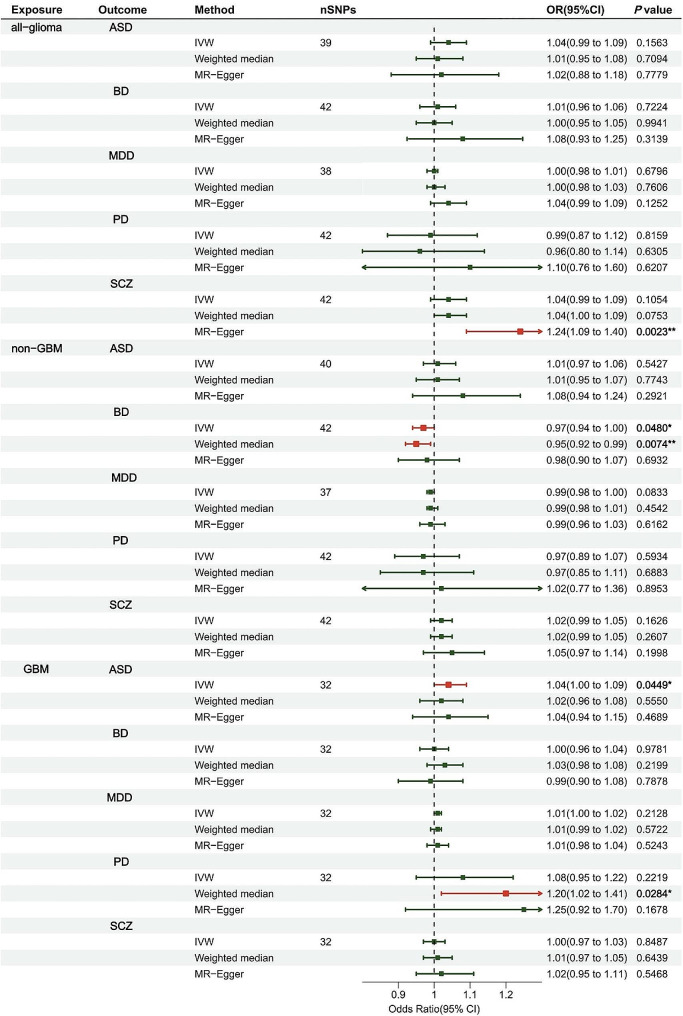
Forrest plot of multivariable MR estimates for the causal associations between glioma and psychiatric disorders Results are reported as odds ratios (OR) and 95% CIs per logOR unit change in the exposure. *: suggestive association (0.01 < *P* < 0.05)

We investigated whether genetically predicted glioma are associated the risk of the 5 psychiatric diseases by using reverse MR analysis. We found a suggestive causal relationship between genetically predicted GBM and an increased risk of ASD (OR = 1.04, 95% CI = 1.00-1.09, *P* = 0.045), and between genetically predicted non-GBM and decreased risk of BD (OR = 0.97, 95% CI = 0.94-1.00, *P* = 0.048). However, leave-one-out analysis indicated that the causal effect of GBM on ASD and that of non-GBM on BD could be driven by particular SNPs. The causality was not observed after the exclusion of the dominant SNPs (Fig. 2; Table 2 and Supplementary Figs. 16–30).

**Table 2:** Sensitivity analyses including weighted median estimator, MR-Egger regression, Cochran’s Q statistic and MR-Egger intercept test for the causal association between glioma and psychiatric disorders

			Weighted median estimator				MR-Egger				Heterogeneity (*P*)	Pleiotropy (*P*)
Exposure	Outcome	SNPs	*P*	OR	Lower 95%CI	Upper 95%CI	*P*	OR	Lower 95%CI	Upper 95%CI		
all-glioma	PD	42	0.631	0.957	0.800	1.144	0.621	1.099	0.758	1.596	0.819	0.542
	ASD	39	0.709	1.013	0.946	1.084	0.778	1.022	0.882	1.183	0.567	0.846
	SCZ	42	0.075	1.044	0.996	1.095	0.002^**^	1.235	1.088	1.404	0.000^**^	0.007^**^
	BD	42	0.994	1.000	0.950	1.054	0.319	1.077	0.932	1.245	0.000^**^	0.351
	MDD	38	0.761	1.003	0.982	1.025	0.125	1.038	0.991	1.087	0.361	0.078
GBM	PD	32	0.028	1.198	1.019	1.408	0.168	1.249	0.918	1.700	0.226	0.322
	ASD	32	0.555	1.018	0.960	1.079	0.469	1.040	0.937	1.154	0.878	0.921
	SCZ	32	0.644	1.009	0.971	1.048	0.547	1.024	0.948	1.106	0.146	0.564
	BD	32	0.220	1.029	0.983	1.078	0.788	0.987	0.899	1.084	0.013^*^	0.760
	MDD	32	0.572	1.005	0.987	1.025	0.524	1.011	0.978	1.044	0.450	0.879
non-GBM	PD	42	0.688	0.973	0.851	1.111	0.895	1.020	0.765	1.358	0.902	0.744
	ASD	40	0.774	1.008	0.953	1.067	0.292	1.078	0.939	1.238	0.002^**^	0.366
	SCZ	42	0.261	1.019	0.986	1.053	0.200	1.054	0.974	1.142	0.029^*^	0.380
	BD	42	0.007^**^	0.951	0.916	0.987	0.693	0.983	0.901	1.072	0.024^*^	0.782
	MDD	37	0.454	0.994	0.978	1.010	0.616	0.991	0.959	1.025	0.321	0.902

PD = panic disorder, ASD = autism spectrum disorder, SCZ = schizophrenia, BD = bipolar disorder, MDD = major depressive disorder

* Showed significant differences *P* < 0.05, ** Bonferroni corrected *P* < 0.01

Heterogeneity was examined by using Cochran’s Q statistic

Pleiotropy was assessed with MR-Egger intercept test

## Discussion

Until now, etiological factors for glioma have not been fully understood. By using the two-sample bi-directional MR analysis, we investigated the causal relationship between genetically predicted five psychiatric disorders and glioma risk. In the current study, we identified the genetic predisposition to SCZ increased the risk of non-GBM glioma, whereas genetically predicted MDD, BD, ASD and PD were not associated with glioma risk. Our findings provide evidence for the etiological basis of glioma and implicate the necessity for medical professional to be cautious for the occurrence of non-GBM glioma in patients with a history of SCZ.

The susceptibility of patients with SCZ to malignancies has been a subject of longstanding debate. However, accumulating evidence indicates that the causal relationship between SCZ and cancer risk is contingent upon the specific disease context. In individuals with SCZ, there is an elevated incidence of breast cancer, ovarian cancer, and thyroid cancer, while a reverse association has been reported for melanoma and bladder cancer [18–20]. The association between SCZ and glioma has been inconclusive. One assumption posits that SCZ exerts a tumor-suppressive effect on glioma. This hypothesis relies on a limited number of observational studies and the underlying assumption that molecular alterations in shared genes and their regulatory networks have contrasting roles in the development of SCZ and glioma [21, 22]. However, our results did not support the protective effect of SCZ against glioma. We found that the causal effect of SCZ on glioma was differently related to glioma subtypes. Specifically, SCZ was found to increase the risk of non-GBM glioma (OR = 1.13, 95% CI: 1.03–1.23, *P* = 0.0096), while no significant influence was observed for GBM. The distinct causal relationship between SCZ and glioma subtypes is likely attributed to connectomic and genetic features specific to certain brain regions. SCZ has been well-recognized as a neurodevelopmental abnormality, which frequently disrupts highly connected hub nodes that functionally integrate anatomically disparate neural systems in brain network. Comprehensive studies on topology of the normative connectome and the anatomy of brain disorders demonstrated that SCZ lesion-concentrated hubs exhibit a non-random spatial distribution, with a notable preference in areas such as anterior cingulate, medial frontal and parahippocampal regions [23, 24]. Meanwhile, recent works also revealed that brain network features are associated with gliomagenesis. Cerebral regions with highly functional hubness are more vulnerable for the occurrence of glioma, partially due to their elevated metabolic turnover. The vibrant energetic demands and increased reactive oxygen species generated by metabolic stress enhance the likelihood of oncogenesis in these regions [25]. In addition, the combined neuroimaging and transcriptomic analysis unveiled a grade-specific pattern of regional susceptibility to gliomas. The constructed grade-related expression map demonstrated that genes overexpressed in GBM are preferentially enriched in parietal and occipital cortices. By contrast, genes overexpressed in non-GBM are found to be expressed in hub nodes such as anterior cingulate, motor, parahippocampal and entorhinal regions, which are commonly involved by SCZ [26]. The accumulation of genetic aberrations specific to non-GBMs might guide the development of gliomas in brain regions frequented by SCZ.

MDD is a major mental illness that leads to disabilities. Chronic depression has been found to undermine immune response through the disturbed function of hypothalamic-pituitary-adrenal axis and elevated levels of proinflammatory cytokines, which also accounts for the development and progression of various cancers [27]. Although observational studies have indicated an association between MDD and glioma, the causal relationship remains inconclusive due to inherent methodological biases and challenges in establishing the chronological order of events [28, 29]. However, our MR showed that genetic predisposition to MDD does not confer an increased risk for glioma.

Our study has several strengths. To the best of our knowledge, this is the first MR study to investigate a causal link between a range of psychiatric disorders and glioma. Secondly, our analyses were based on information from the largest glioma GWAS datasets to date that compose more than 30,000 participants. In addition, the leverage of multiple germline genetic variants as proxies for psychiatric disorders reduces potential bias in conventional epidemiological studies. Moreover, we performed various sensitivity analyses, such as exploring horizontal pleiotropy by using MR-PRESSO approach, to support the robust causal inference [14]. However, several limitations should be acknowledged. Firstly, analyses in this study investigated GWAS data derived from participants of European ancestry. Validation of our findings in other ethnic groups is warranted. Secondly, the presence of potential confounders, including hidden population stratification, cannot be entirely ruled out, which may introduce bias in the risk associations [30].

In conclusion, by using a comprehensive Mendelian randomization approach, our study contributes evidence supporting the potential causal impact of mental illness on glioma. Specifically, we identified an increased risk of non-GBM glioma associated with schizophrenia. Genetic susceptibility in the overlapping of connectomic distribution of SCZ and non-GBM glioma may account for the association. In term of clinical pratice, it is important for healthcare providers to be vigilant to the potential occurrence of non-GBM glioma in patient with a history of SCZ. Meanwhile, further prospective studies and mechanistic investigations were warranted for a better understanding of the causative effect of SCZ on glioma.

### Electronic supplementary material

Below is the link to the electronic supplementary material.

**Supplementary Table 1** Summary of 8 glioma GWAS datasets

**Supplementary Table 2** Single SNP analysis for the causal association betweenpsychiatric disorders and all-glioma. **Supplementary Table 3** Single SNP analysis for the causal association betweenpsychiatric disorders and GBM. **Supplementary Table 4** Single SNP analysis for the causal association betweenpsychiatric disorders and non-GBM. **Supplementary Table 5** Single SNP analysis for the causal association betweenall-glioma and psychiatric disorders. **Supplementary Table 6** Single SNP analysis for the causal association between GBM and psychiatric disorders. **Supplementary Table 7** Single SNP analysis for the causal association between non-GBM and psychiatric disorders. **Supplementary Table 8** Analysis for the causal association between schizophrenia and non-GBM by using MR-cML methods

**Supplementary Fig. 1** The leave-one-out plot, funnel plot, and scatter plot for the causal association between schizophrenia and non-GBM in the primary analysis. **Supplementary Fig. 2** The leave-one-out plot, funnel plot, and scatter plot for the causal association between of schizophrenia and GBM in the primary analysis. **Supplementary Fig. 3** The leave-one-out plot, funnel plot, and scatter plot for the causal association between schizophrenia and all-glioma in the primary analysis. **Supplementary Fig. 4** The leave-one-out plot, funnel plot, and scatter plot for the causal association between panic disorder and non-GBM in the primary analysis. **Supplementary Fig. 5** The leave-one-out plot, funnel plot, and scatter plot for the causal association between panic disorder and GBM in the primary analysis. **Supplementary Fig. 6** The leave-one-out plot, funnel plot, and scatter plot for the causal association between panic disorder and all-glioma in the primary analysis. **Supplementary Fig. 7** The leave-one-out plot, funnel plot, and scatter plot for the causal association between autistic spectrum disorder and non-GBM in the primary analysis. **Supplementary Fig. 8** The leave-one-out plot, funnel plot, and scatter plot for the causal association between autistic spectrum disorder and GBM in the primary analysis. **Supplementary Fig. 9** The leave-one-out plot, funnel plot, and scatter plot for the causal association between autistic spectrum disorder and all-glioma in the primary analysis. **Supplementary Fig. 10** The leave-one-out plot, funnel plot, and scatter plot for the causal association between bipolar disorder and non-GBM in the primary analysis. **Supplementary Fig. 11** The leave-one-out plot, funnel plot, and scatter plot for the causal association between bipolar disorder and GBM in the primary analysis. **Supplementary Fig. 12** The leave-one-out plot, funnel plot, and scatter plot for the causal association between bipolar disorder and all-glioma in the primary analysis. **Supplementary Fig. 13** The leave-one-out plot, funnel plot, and scatter plot for the causal association between major depressive disorder and non-GBM in the primary analysis. **Supplementary Fig. 14** The leave-one-out plot, funnel plot, and scatter plot for the causal association between major depressive disorder and GBM in the primary analysis. **Supplementary Fig. 15** The leave-one-out plot, funnel plot, and scatter plot for the causal association between major depressive disorder and all-glioma in the primary analysis. **Supplementary Fig. 16** The leave-one-out plot, funnel plot, and scatter plot for the association between non-GBM and schizophrenia in the reverse MR analysis. **Supplementary Fig. 17** The leave-one-out plot, funnel plot, and scatter plot for the causal association between GBM and schizophrenia in the reverse MR analysis. **Supplementary Fig. 18** The leave-one-out plot, funnel plot, and scatter plot for the causal association between all-glioma and schizophrenia in the reverse MR analysis. **Supplementary Fig. 19** The leave-one-out plot, funnel plot, and scatter plot for the causal association between non-GBM and panic disorder in the reverse MR analysis. **Supplementary Fig. 20** The leave-one-out plot, funnel plot, and scatter plot for the causal association between GBM and panic disorder in the reverse MR analysis. **Supplementary Fig. 21** The leave-one-out plot, funnel plot, and scatter plot for the association between all-glioma and panic disorder in the reverse MR analysis. **Supplementary Fig. 22** The leave-one-out plot, funnel plot, and scatter plot for the causal association between non-GBM and autistic spectrum disorder in the reverse MR analysis. **Supplementary Fig. 23** The leave-one-out plot, funnel plot, and scatter plot for the causal association between GBM and autistic spectrum disorder in the reverse MR analysis. **Supplementary Fig. 24** The leave-one-out plot, funnel plot, and scatter plot for the causal association between all-glioma autistic spectrum disorder in the reverse MR analysis. **Supplementary Fig. 25** The leave-one-out plot, funnel plot, and scatter plot for the causal association between non-GBM and bipolar disorder in the reverse MR analysis. **Supplementary Fig. 26** The leave-one-out plot, funnel plot, and scatter plot for the causal association between GBM and bipolar disorder in the reverse MR analysis. **Supplementary Fig. 27** The leave-one-out plot, funnel plot, and scatter plot for the causal association between all-glioma and bipolar disorder in the reverse MR analysis. **Supplementary Fig. 28** The leave-one-out plot, funnel plot, and scatter plot for the causal association between non-GBM and major depressive disorder in the reverse MR analysis. **Supplementary Fig. 29** The leave-one-out plot, funnel plot, and scatter plot for the causal association between GBM and major depressive disorder in the reverse MR analysis. **Supplementary Fig. 30** The leave-one-out plot, funnel plot, and scatter plot for the association of all-glioma and major depressive disorder in the reverse MR analysis

## Data Availability

All the custom code and data will be made available upon request to corresponding author.
