# Supplementary material for: Association between psychiatric disorders and glioma risk: evidence from Mendelian randomization analysis
Source: BMC Cancer. 2024 Jan 23;24:118. doi: 10.1186/s12885-024-11865-y (PMC10807081; doi:10.1186/s12885-024-11865-y)
Supplement: Supplementary file 1 — Supplementary Table 1 Summary of 8 glioma GWAS datasets [file 12885_2024_11865_MOESM1_ESM.docx]

**Supplementary Table 1** Summary of 8 glioma GWAS datasets

| **Dataset** | **PubMID** | **non-GBM cases** | **GBM cases** | **All-glioma cases** | **Controls** |
| --- | --- | --- | --- | --- | --- |
| UK-GWAS | 17636416 | 361 | 270 | 631 | 2699 |
| French-GWAS | 21531791 | 993 | 430 | 1423 | 1190 |
| German-GWAS | 26424050 | 415 | 431 | 846 | 1310 |
| MDA-GWAS | 19578367 | 523 | 652 | 1175 | 2236 |
| UCSF- SFAGS | 19578367 | 166 | 511 | 677 | 3940 |
| GliomaScan | 22886559 | 472 | 903 | 1653 | 2725 |
| GICC | 26656478 | 1898 | 2460 | 4564 | 3265 |
| UCSF/Mayo | 19578366 | 992 | 526 | 1519 | 804 |
| Total |  | 5820 | 6183 | 12488 | 18169 |
