# Supplementary material for: Association between psychiatric disorders and glioma risk: evidence from Mendelian randomization analysis
Source: BMC Cancer. 2024 Jan 23;24:118. doi: 10.1186/s12885-024-11865-y (PMC10807081; doi:10.1186/s12885-024-11865-y)

**Supplementary Fig. 1** The leave-one-out plot, funnel plot, and scatter plot for the causal association between schizophrenia and non-GBM in the primary analysis


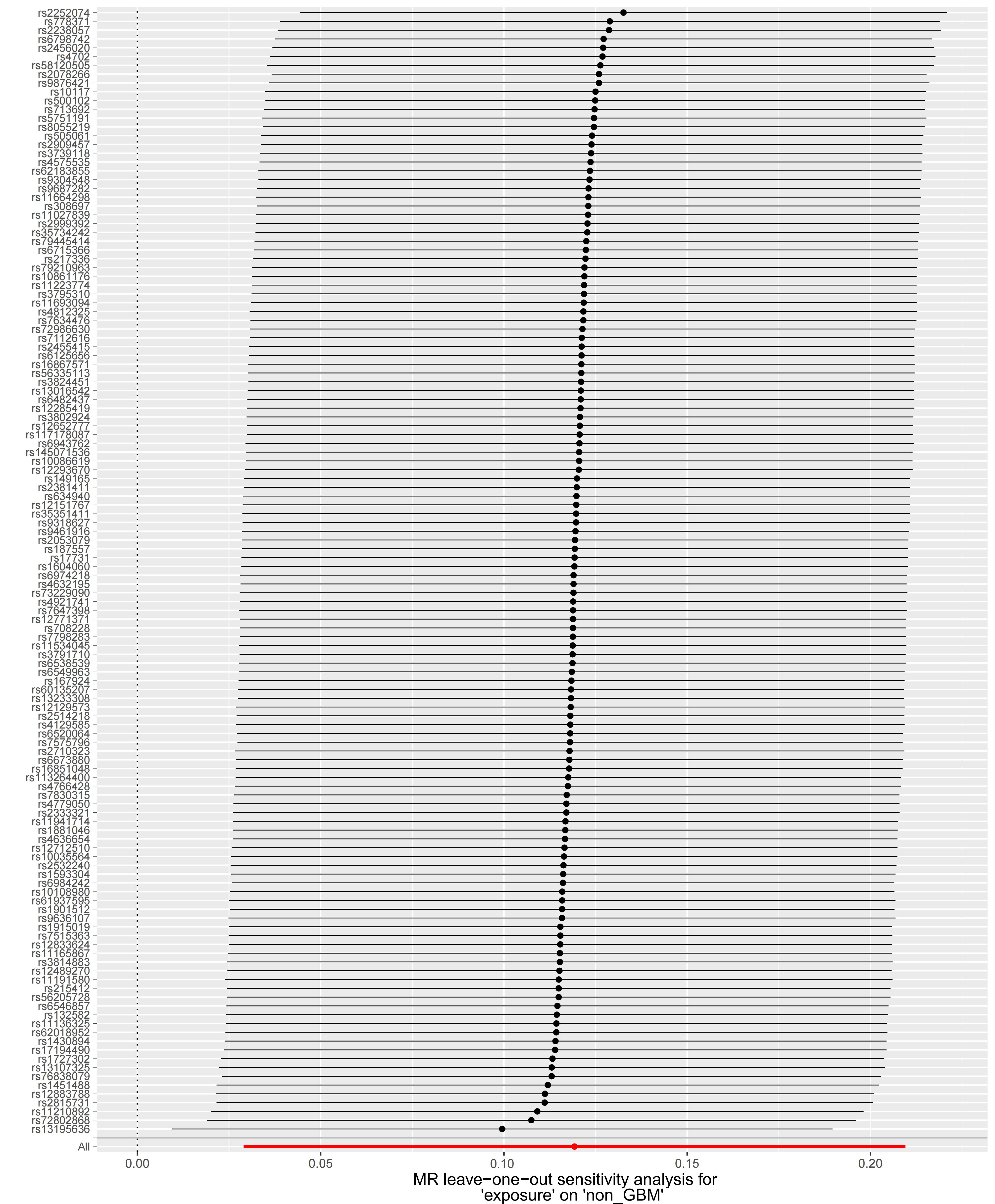


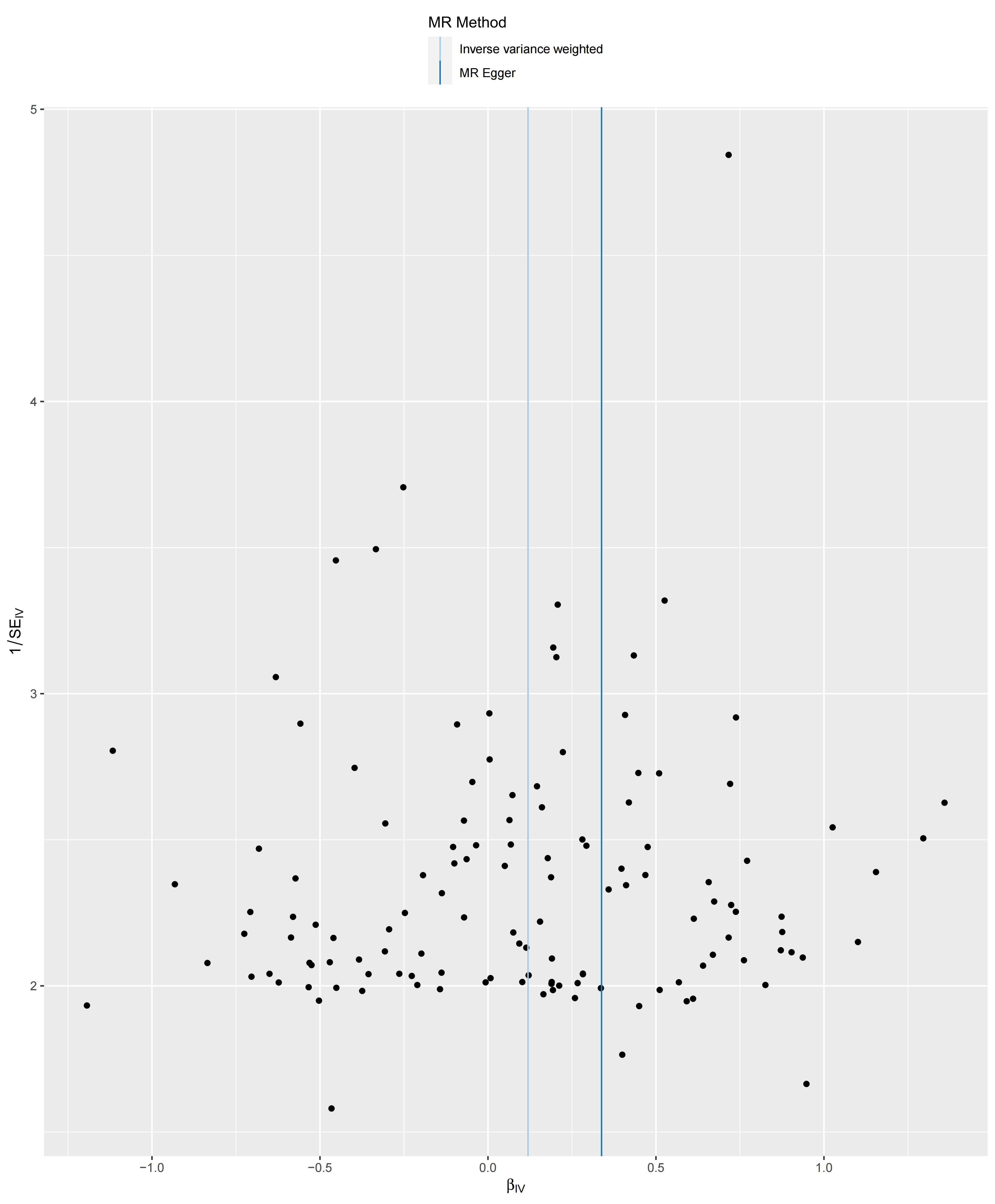


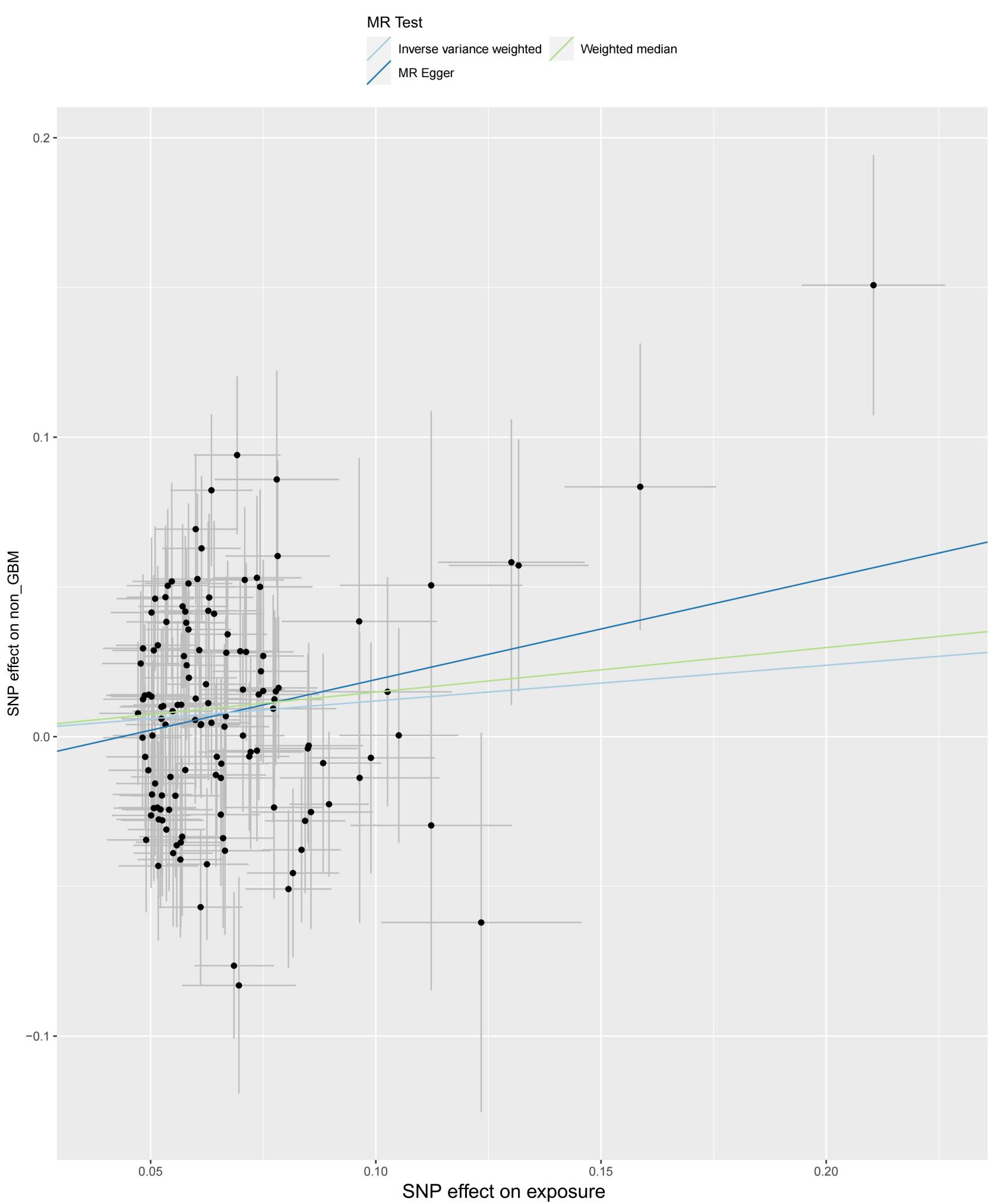


**Supplementary Fig. 2** The leave-one-out plot, funnel plot, and scatter plot for the causal association between of schizophrenia and GBM in the primary analysis


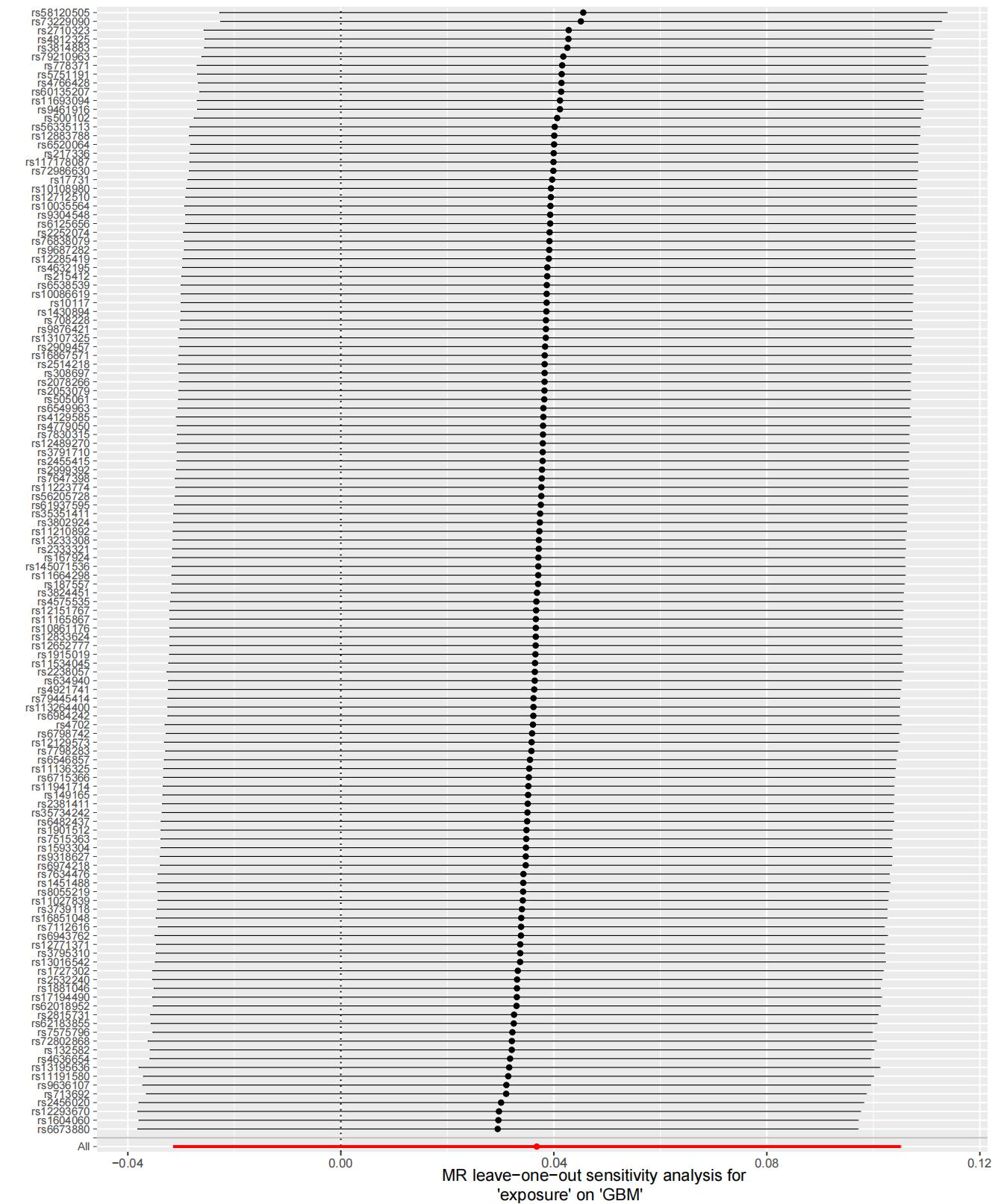


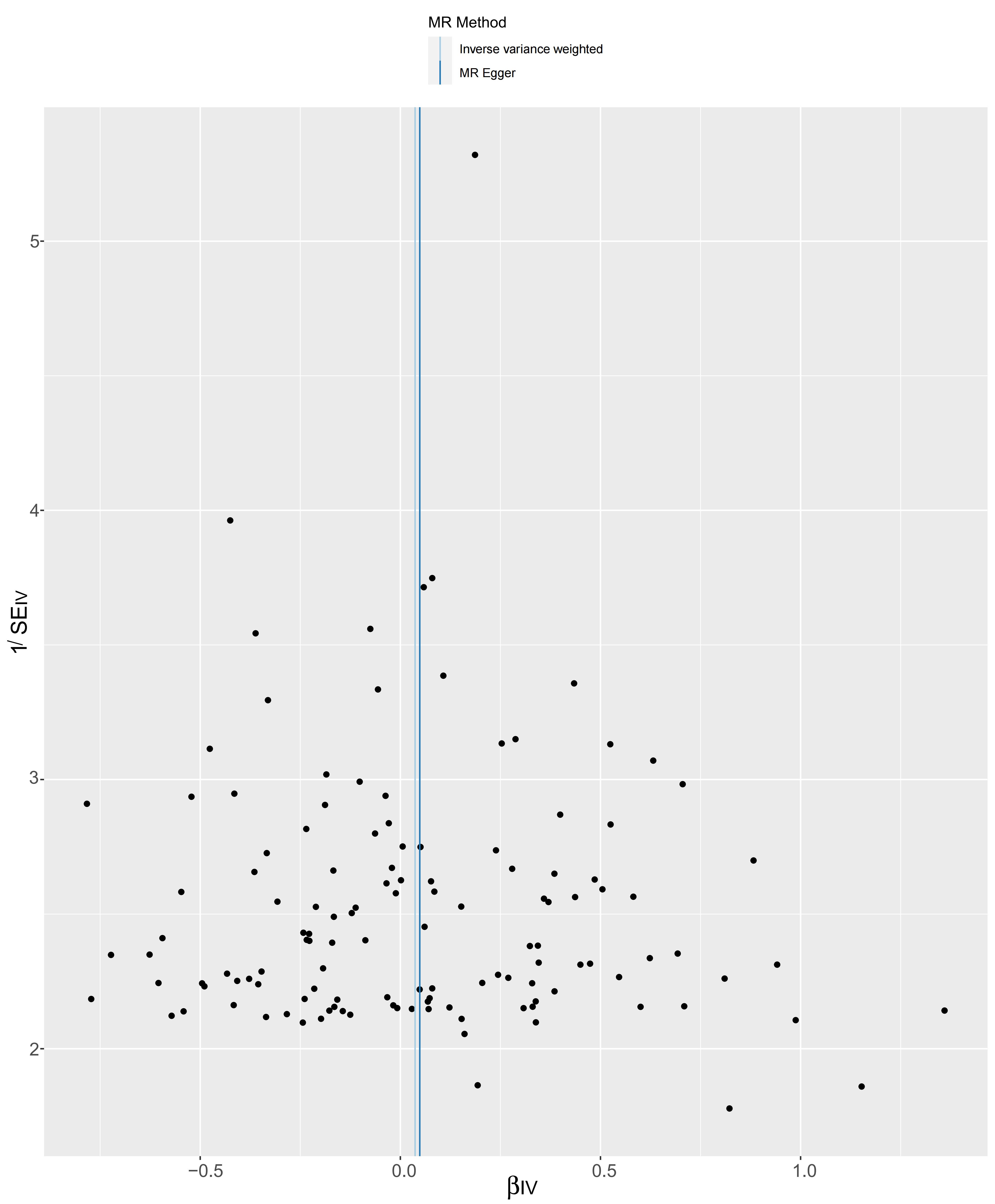


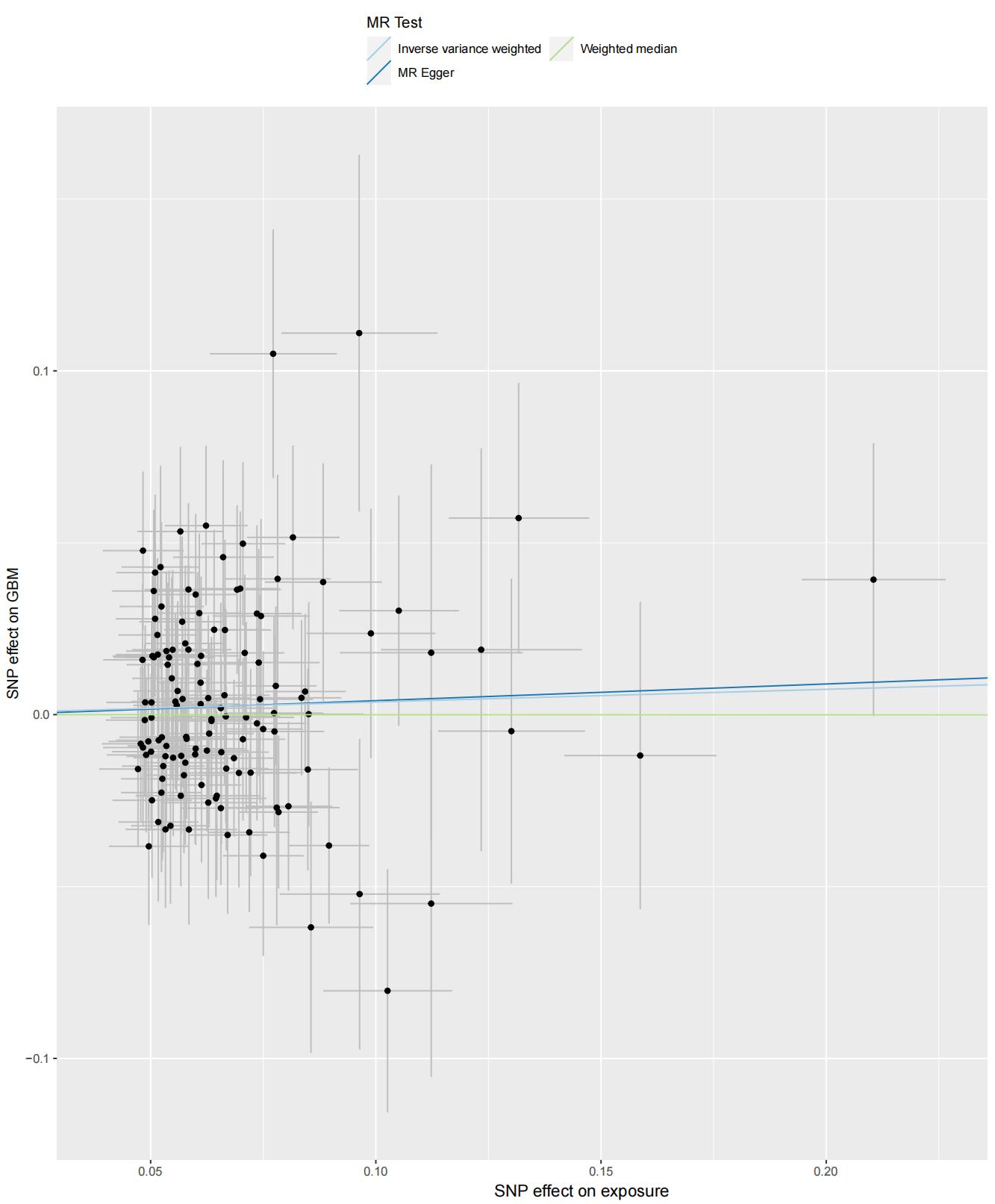


**Supplementary Fig. 3** The leave-one-out plot, funnel plot, and scatter plot for the causal association between schizophrenia and all-glioma in the primary analysis


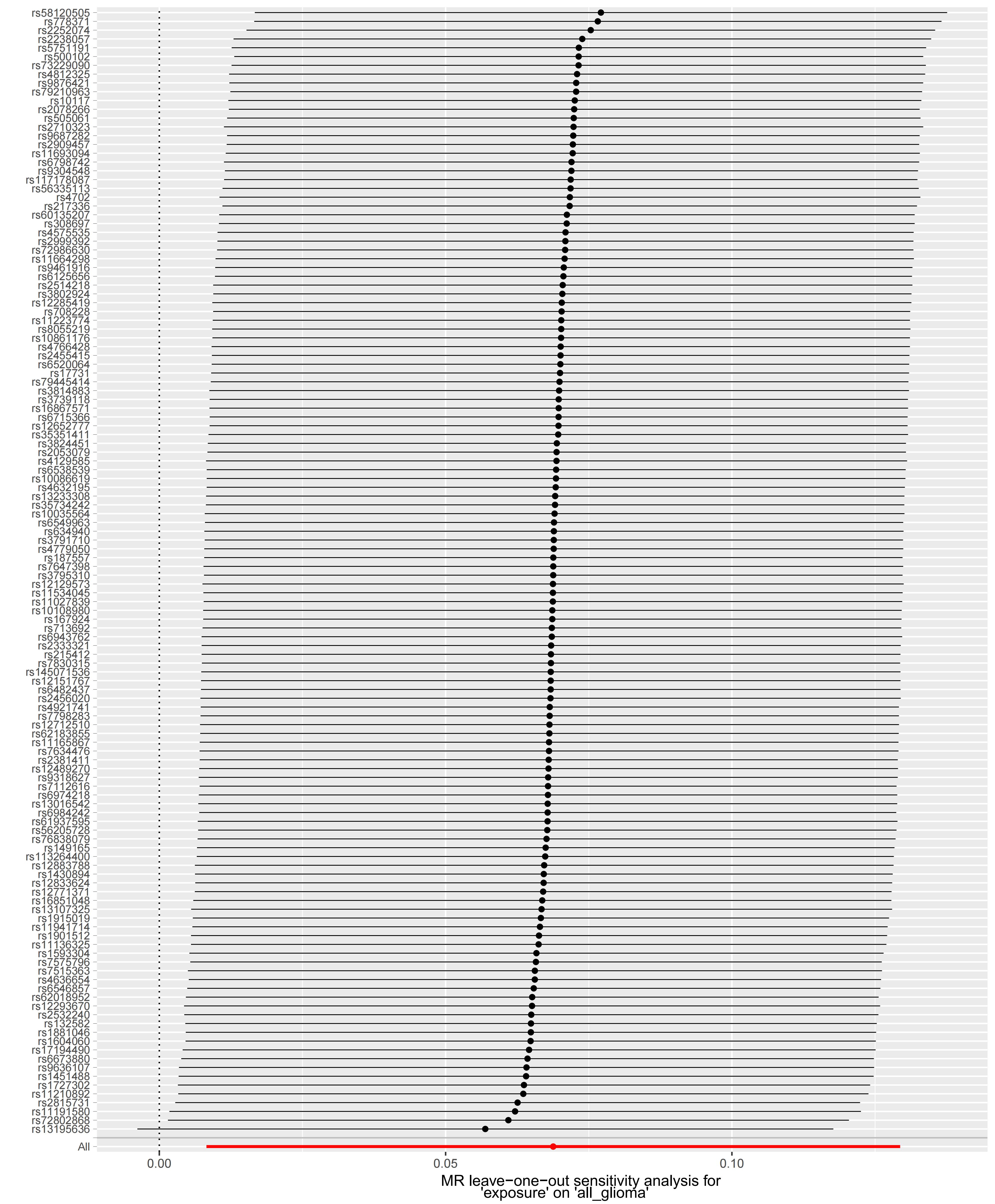


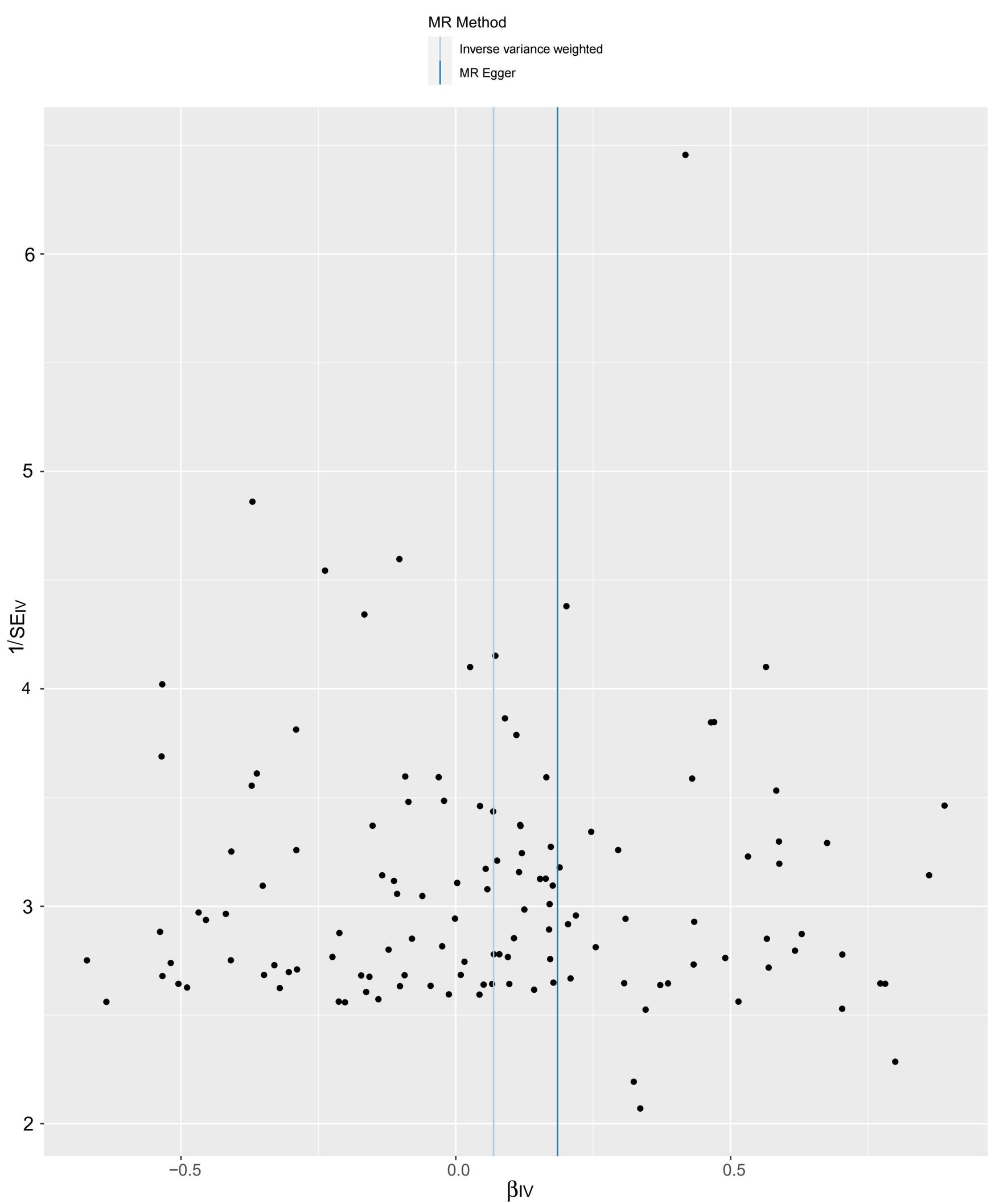


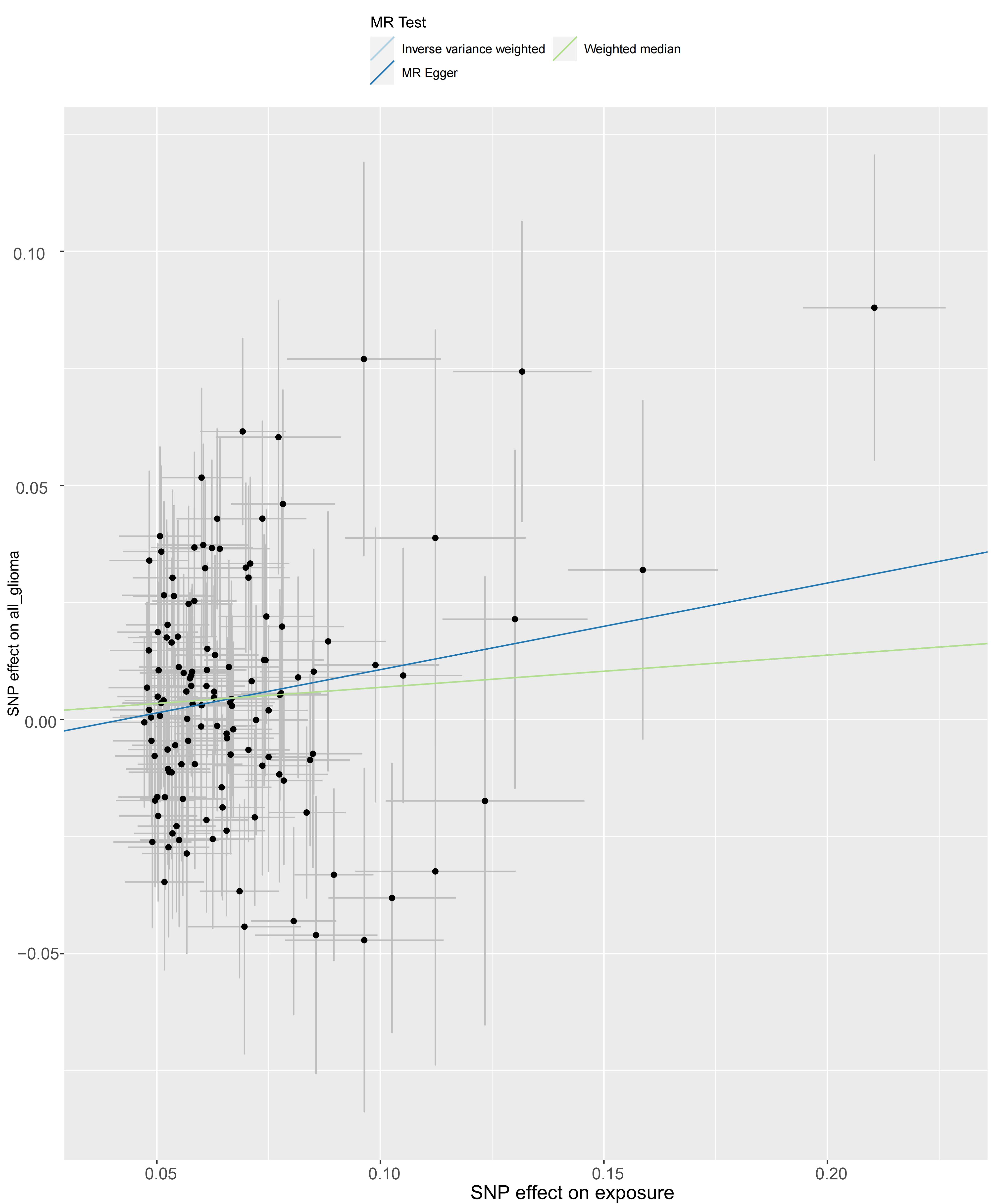


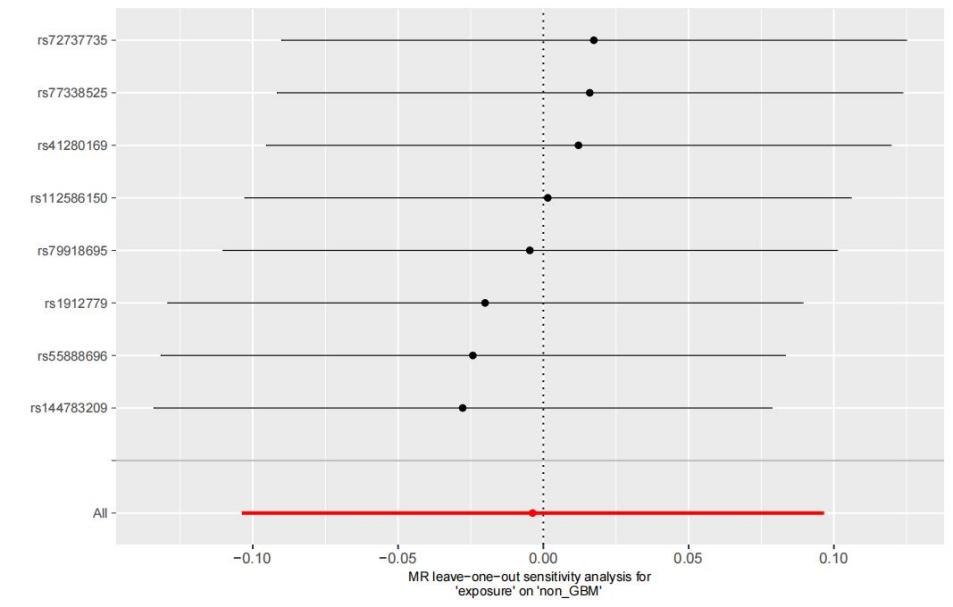
**Supplementary Fig. 4** The leave-one-out plot, funnel plot, and scatter plot for the causal association between panic disorder and non-GBM in the primary analysis


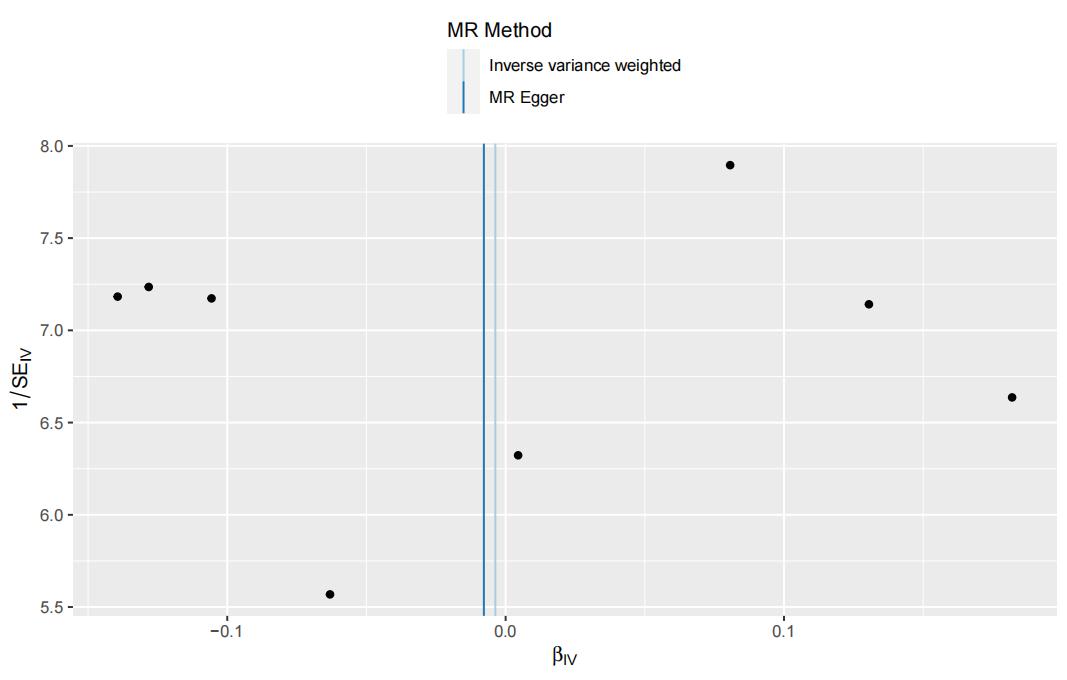


**
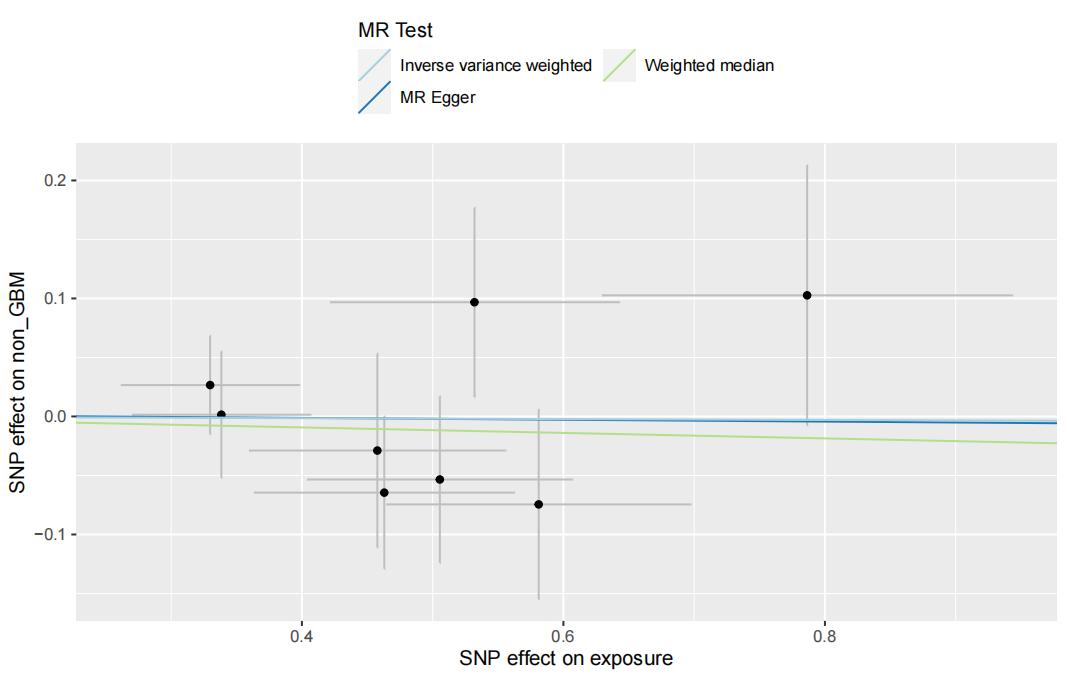
**


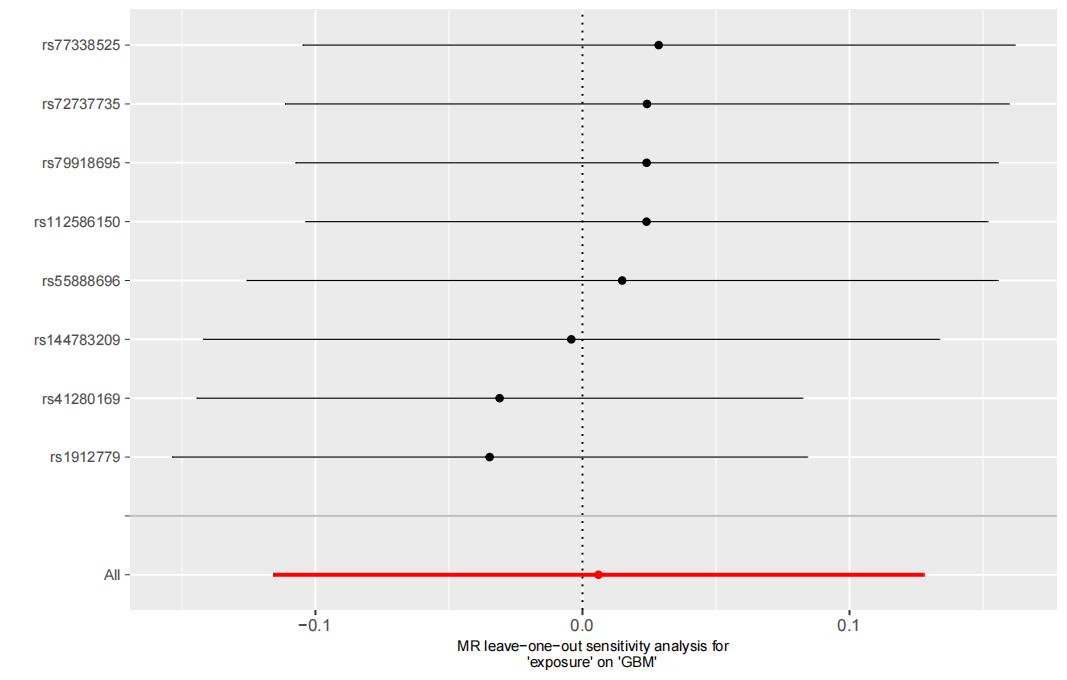
**Supplementary Fig. 5** The leave-one-out plot, funnel plot, and scatter plot for the causal association between panic disorder and GBM in the primary analysis

**
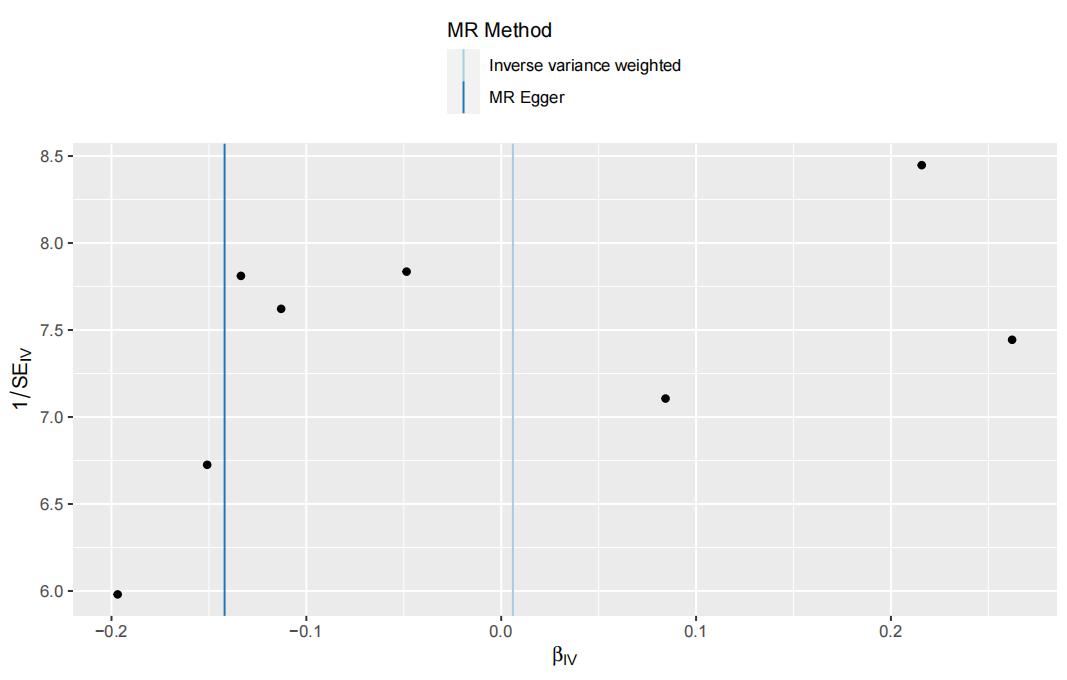
**

**
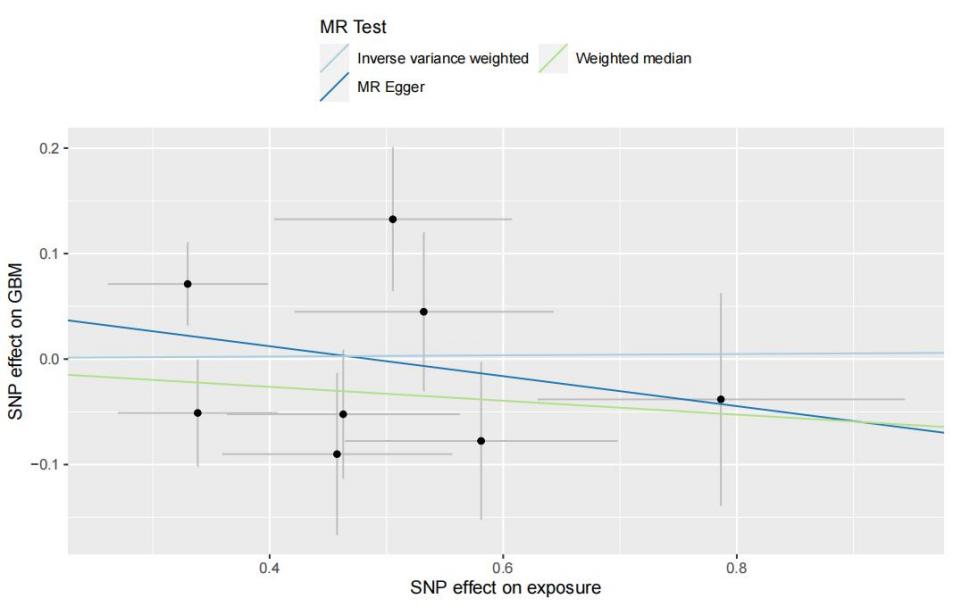
**

**Supplementary Fig. 6** The leave-one-out plot, funnel plot, and scatter plot for the causal association between panic disorder and all-glioma in the primary analysis


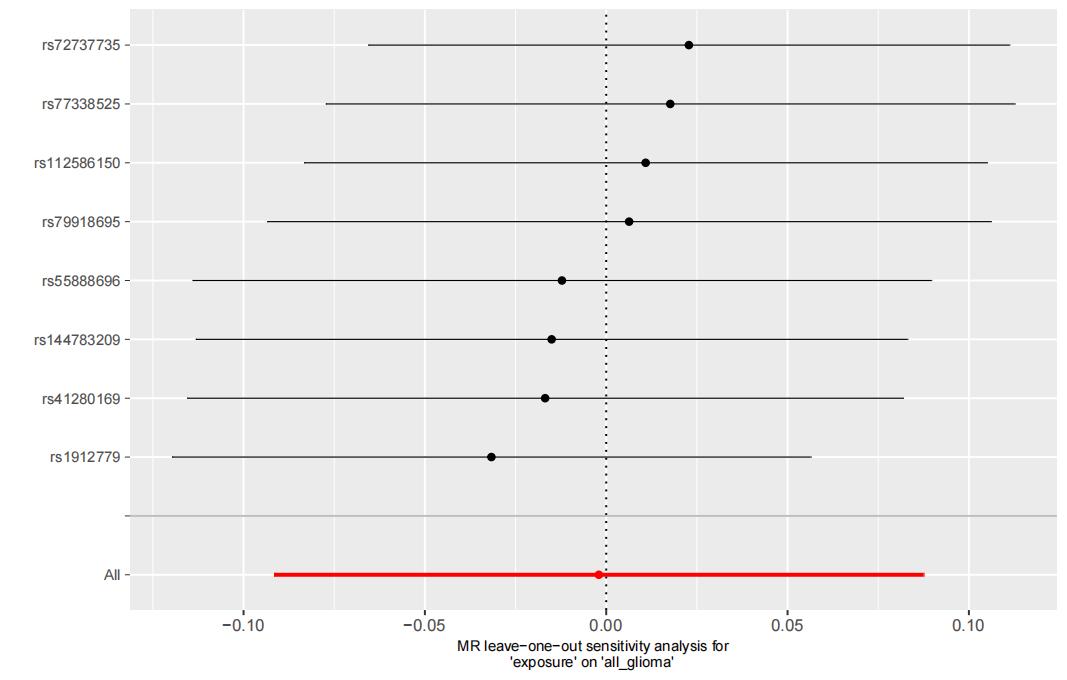


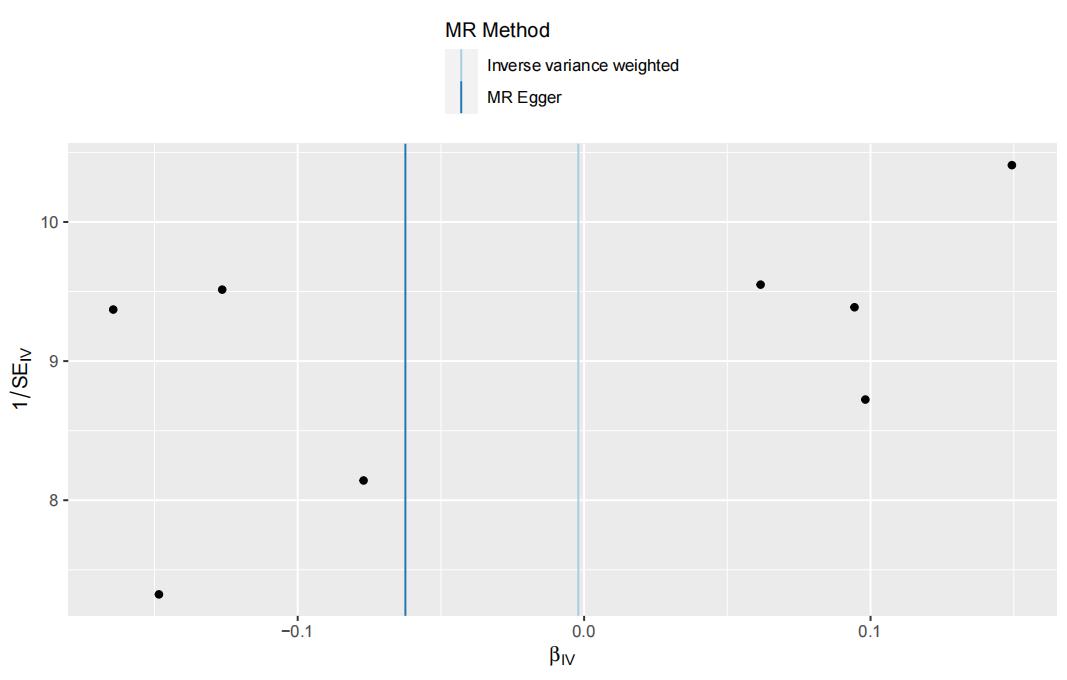


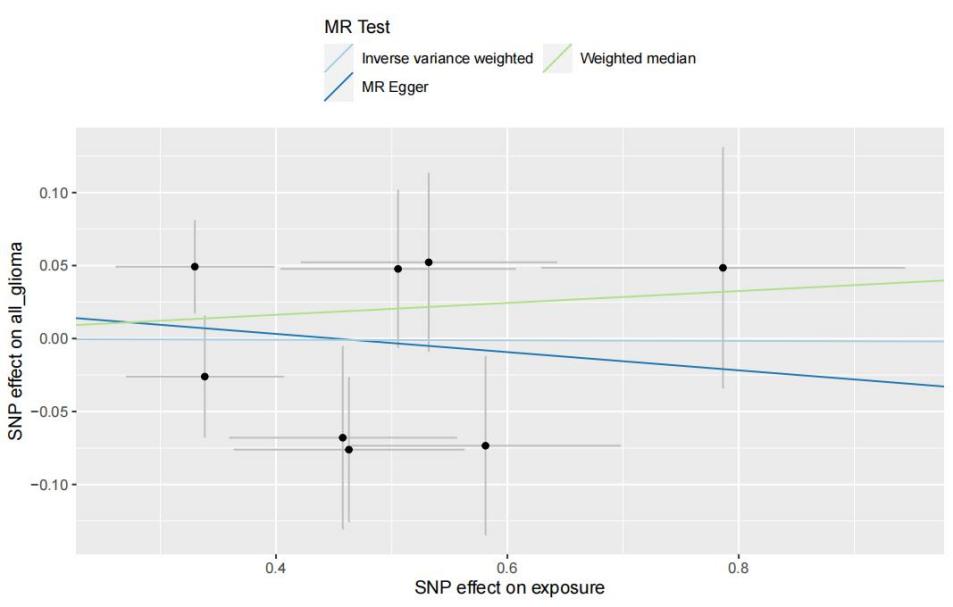


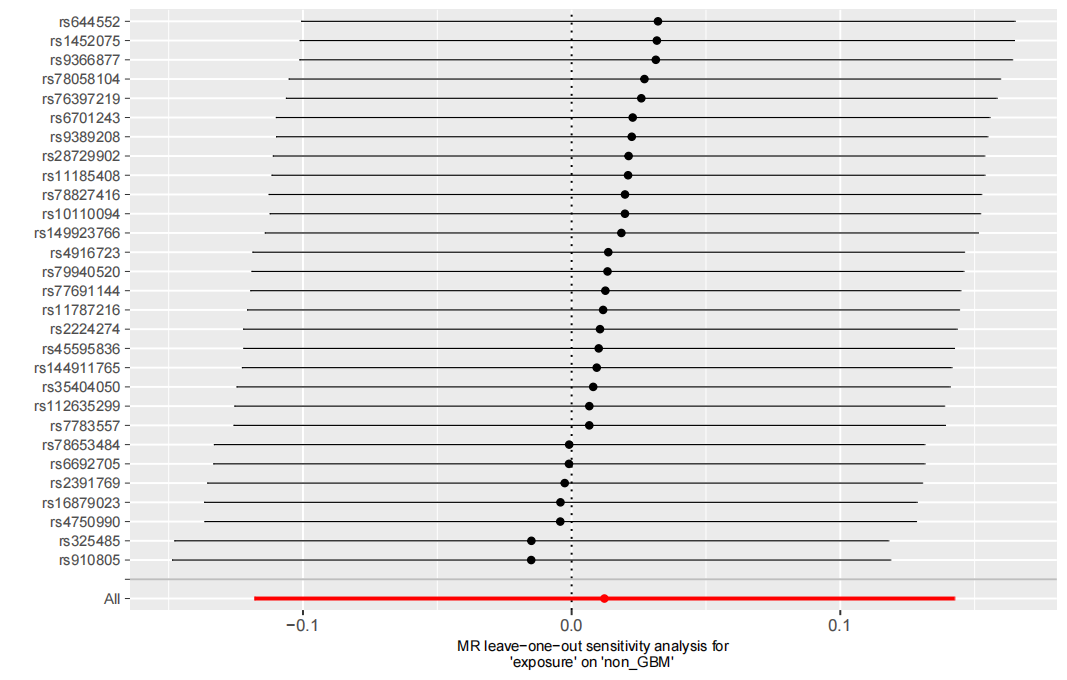
**Supplementary Fig. 7** The leave-one-out plot, funnel plot, and scatter plot for the causal association between autistic spectrum disorder and non-GBM in the primary analysis


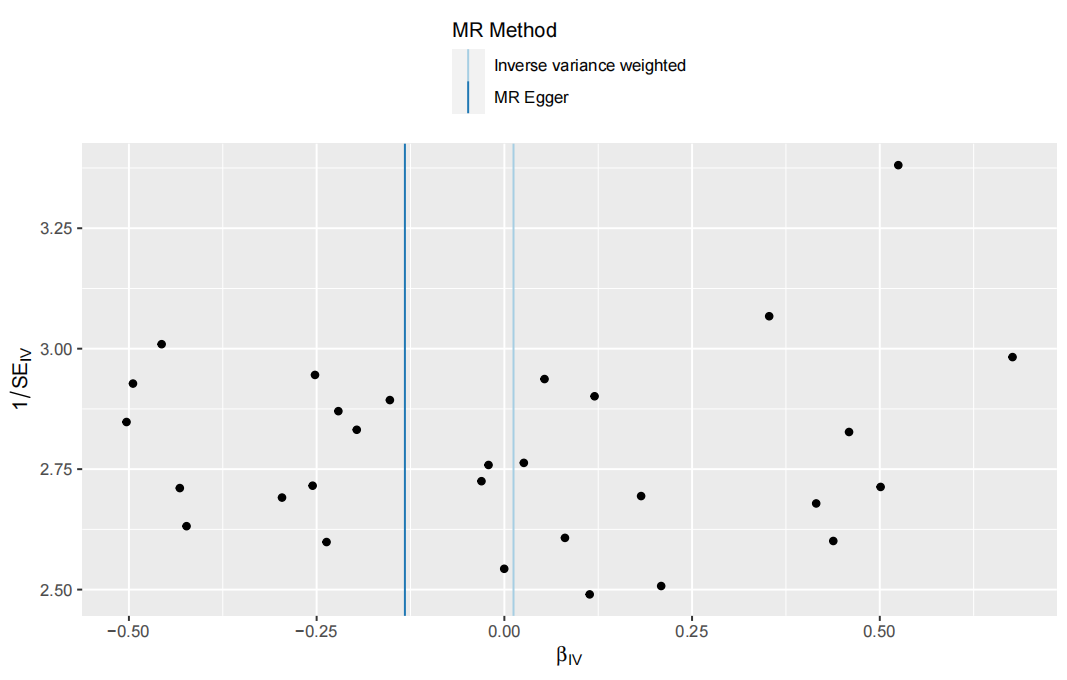


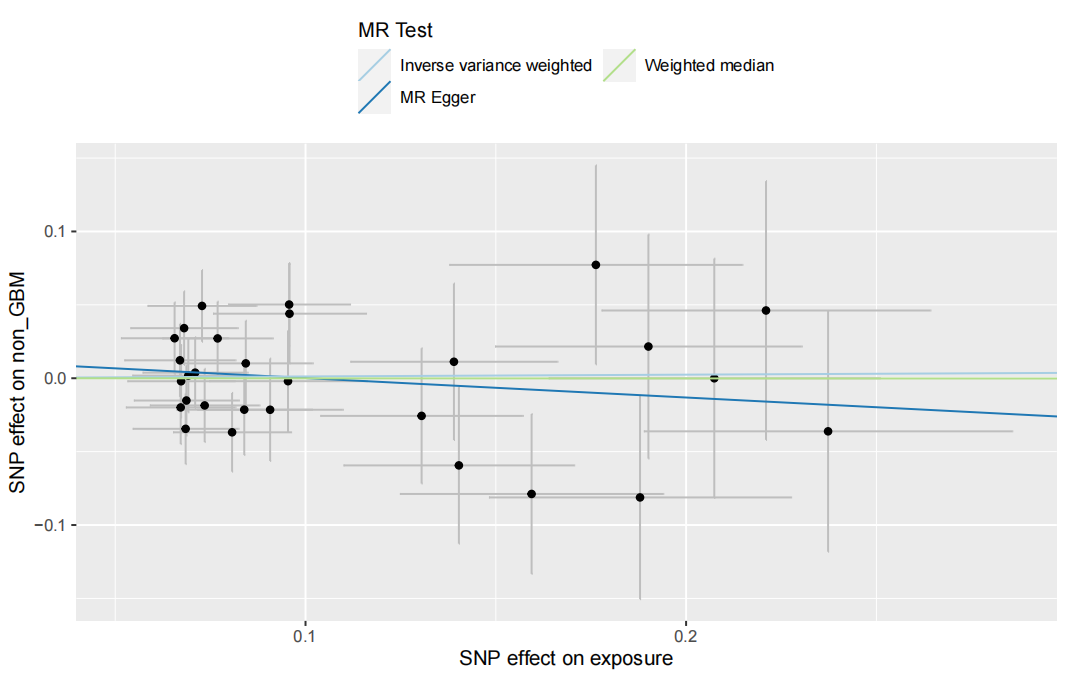


**Supplementary Fig. 8** The leave-one-out plot, funnel plot, and scatter plot for the causal association between autistic spectrum disorder and GBM in the primary analysis


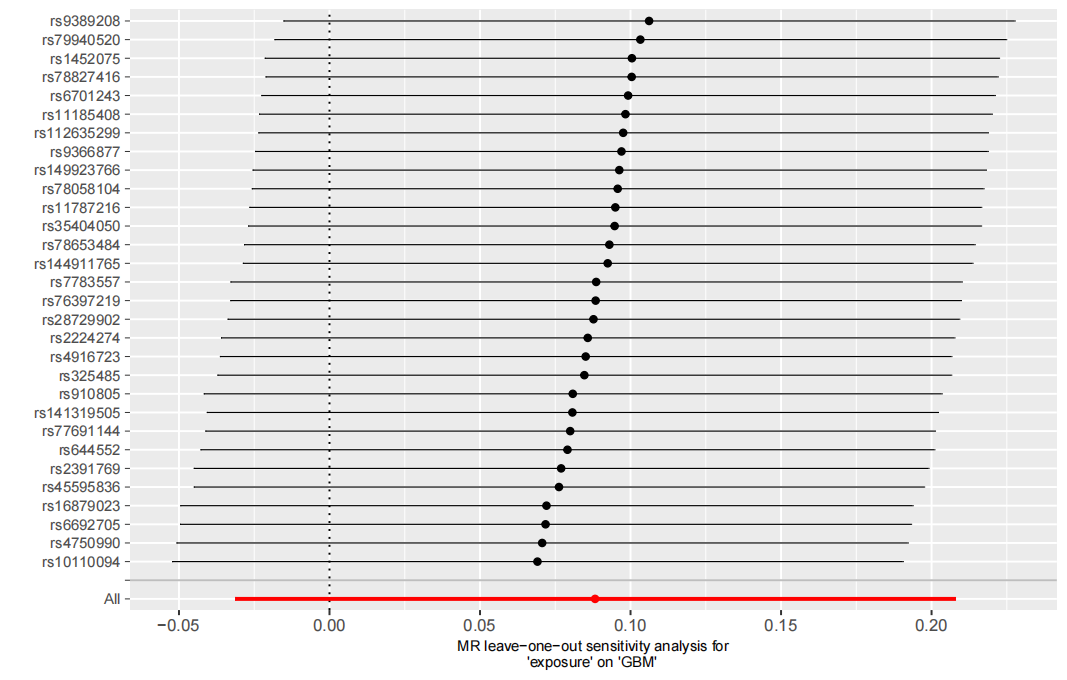


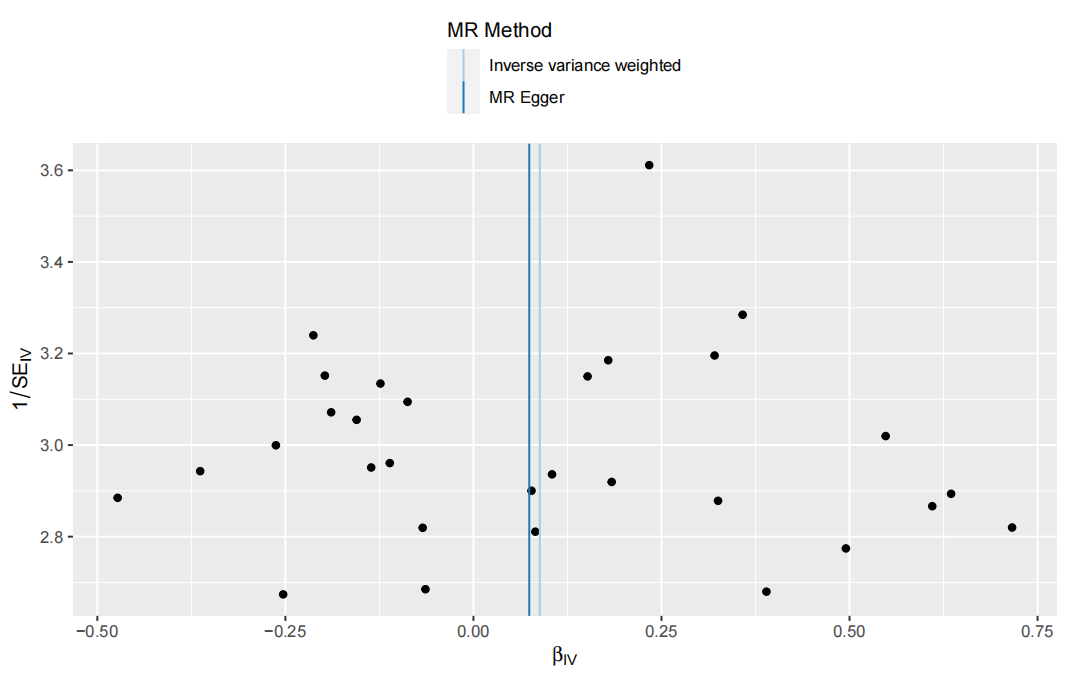


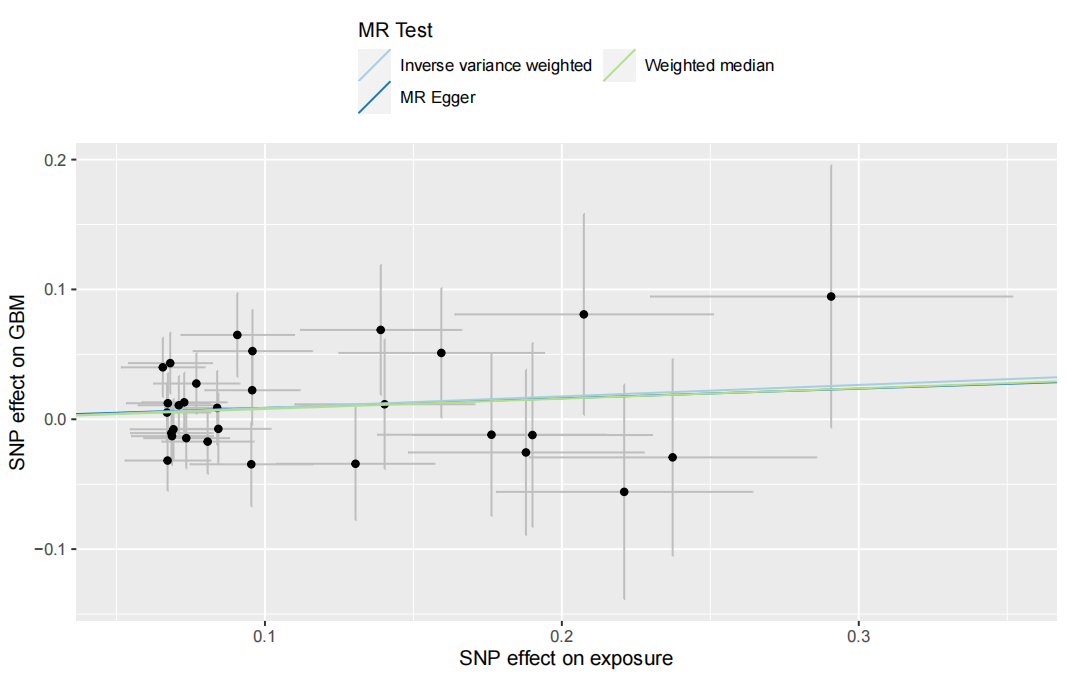


**Supplementary Fig. 9** The leave-one-out plot, funnel plot, and scatter plot for the causal association between autistic spectrum disorder and all-glioma in the primary analysis

**
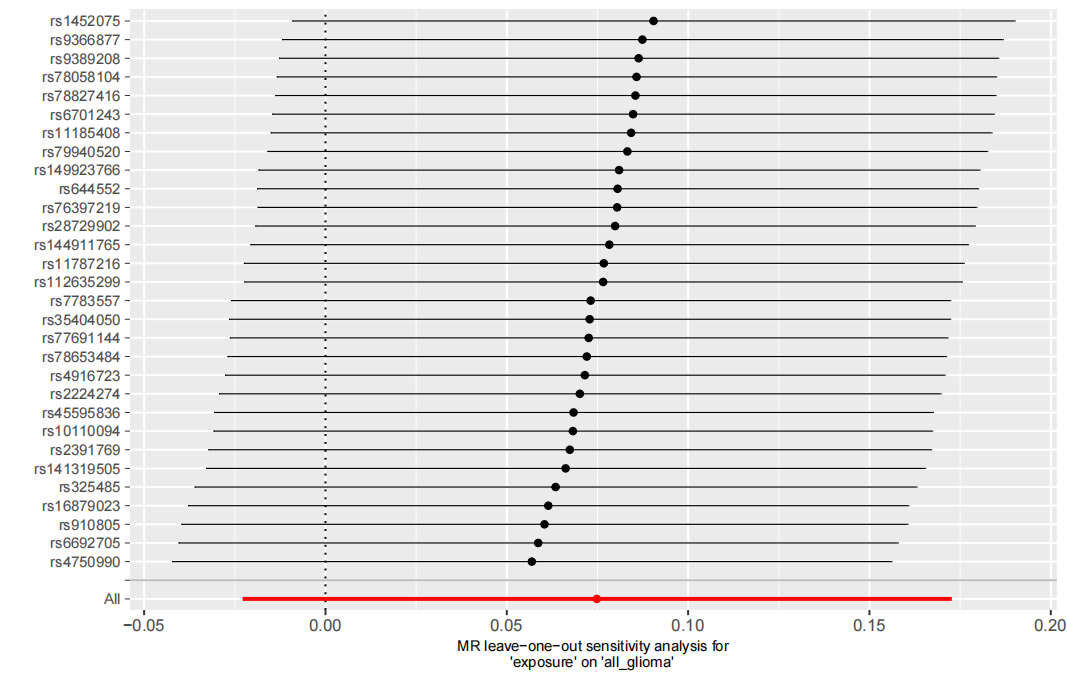
**

**
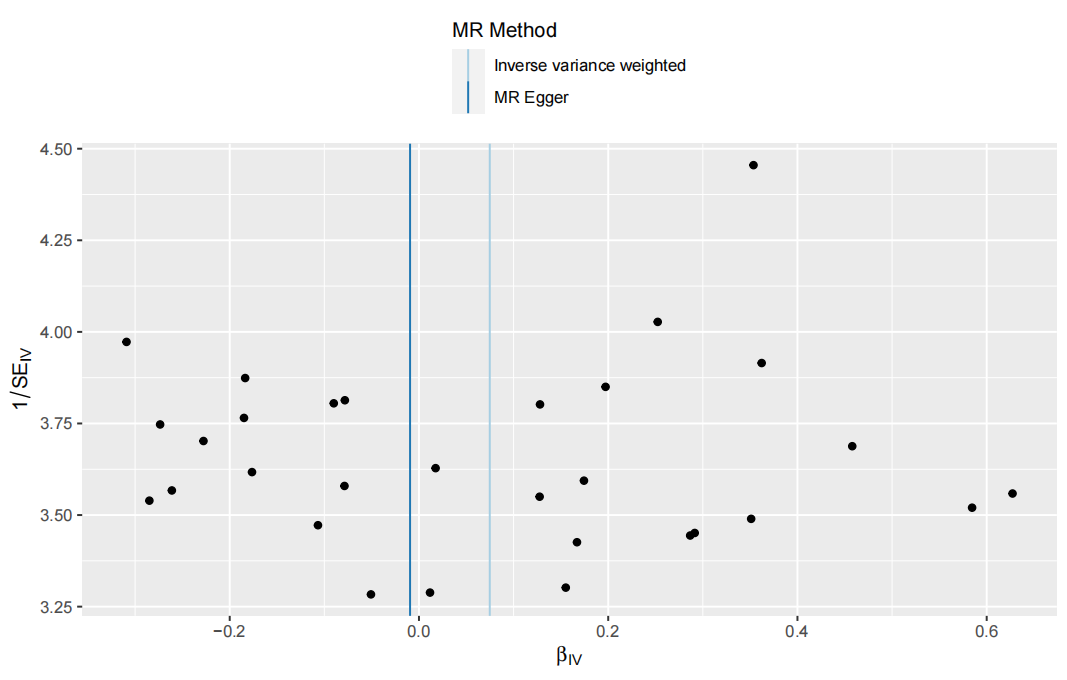
**

**
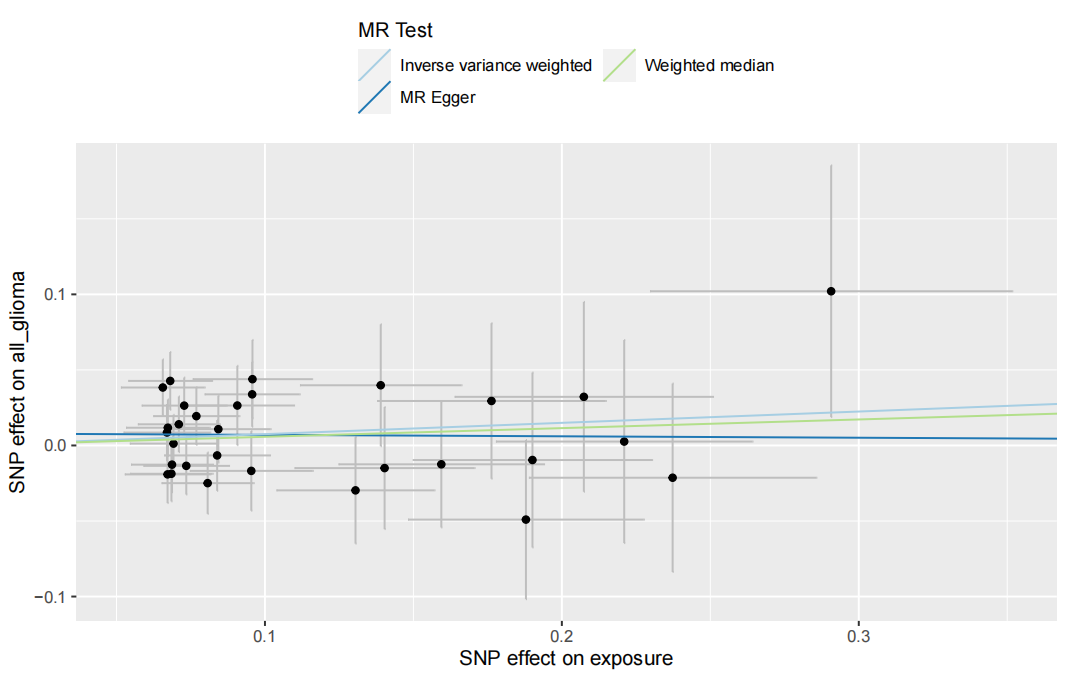
**

**Supplementary Fig. 10** The leave-one-out plot, funnel plot, and scatter plot for the causal association between bipolar disorder and non-GBM in the primary analysis


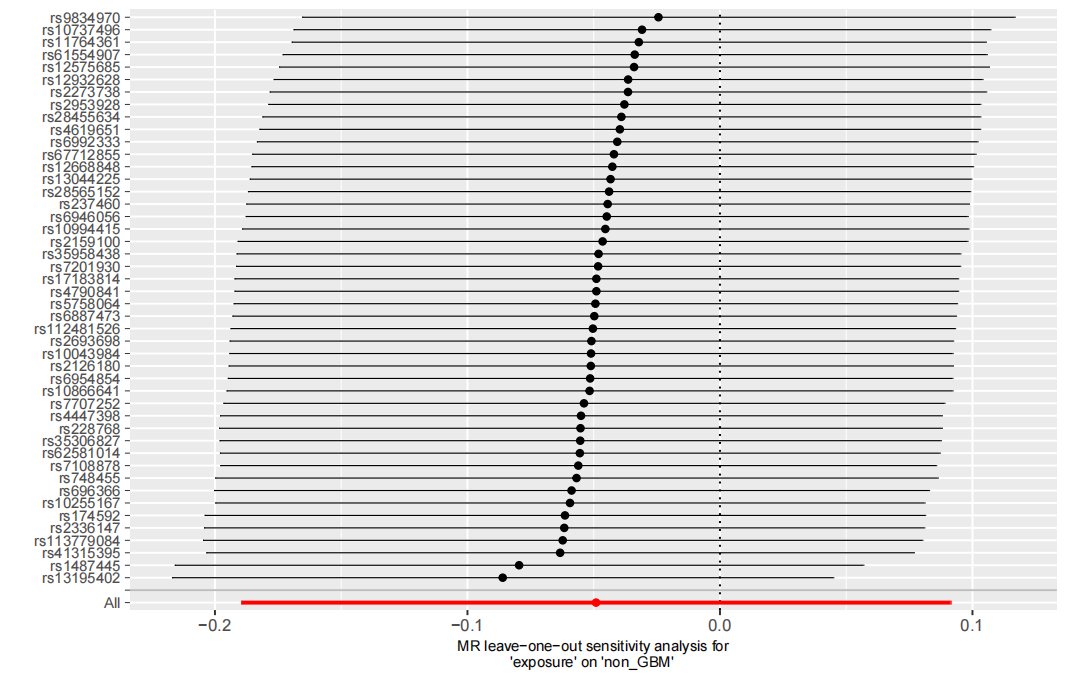


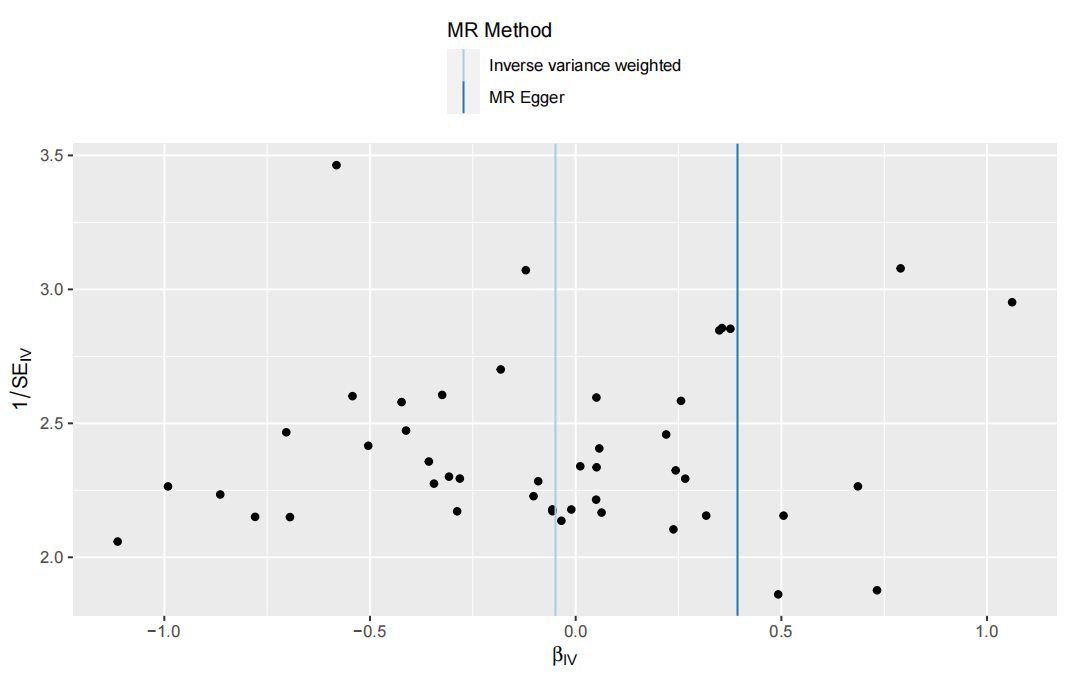


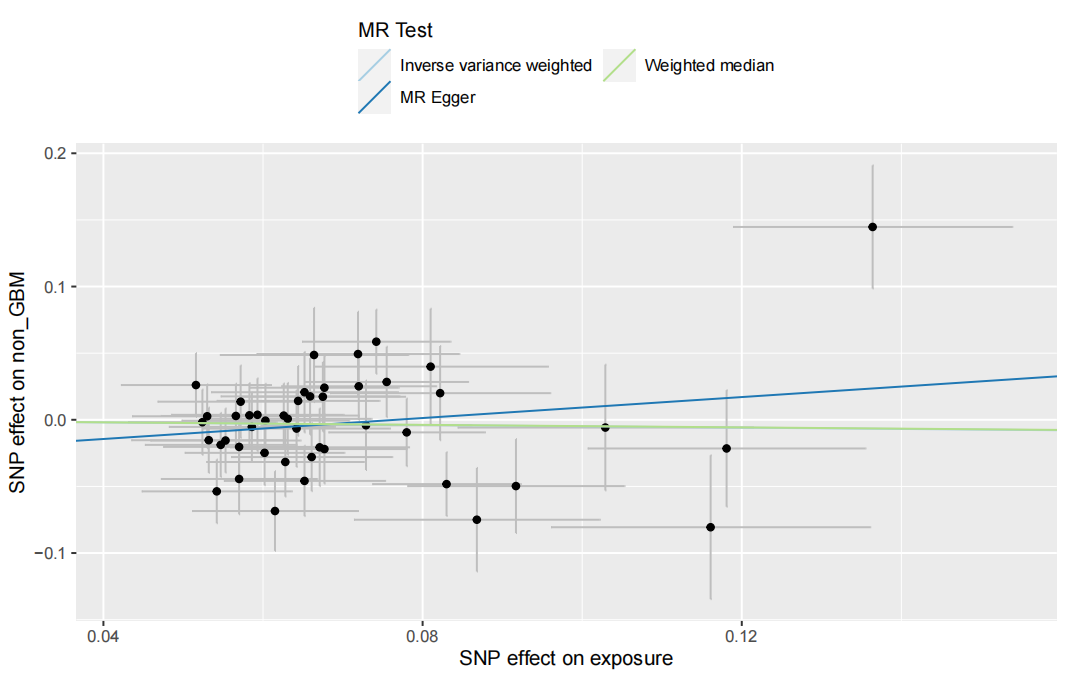


**Supplementary Fig. 11** The leave-one-out plot, funnel plot, and scatter plot for the causal association between bipolar disorder and GBM in the primary analysis


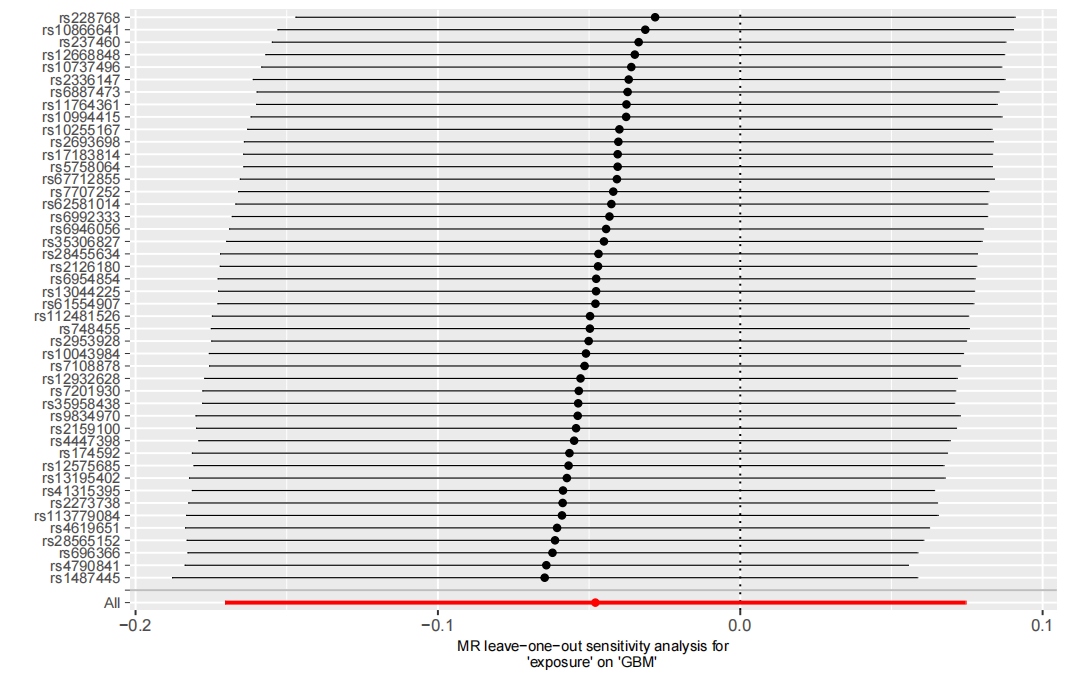


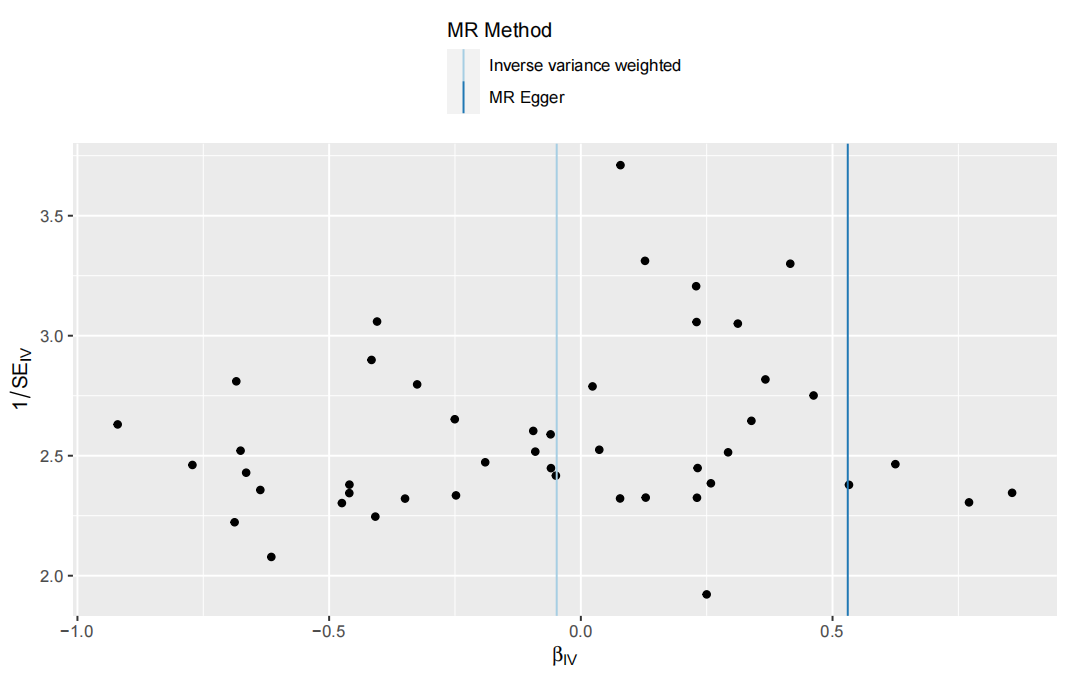


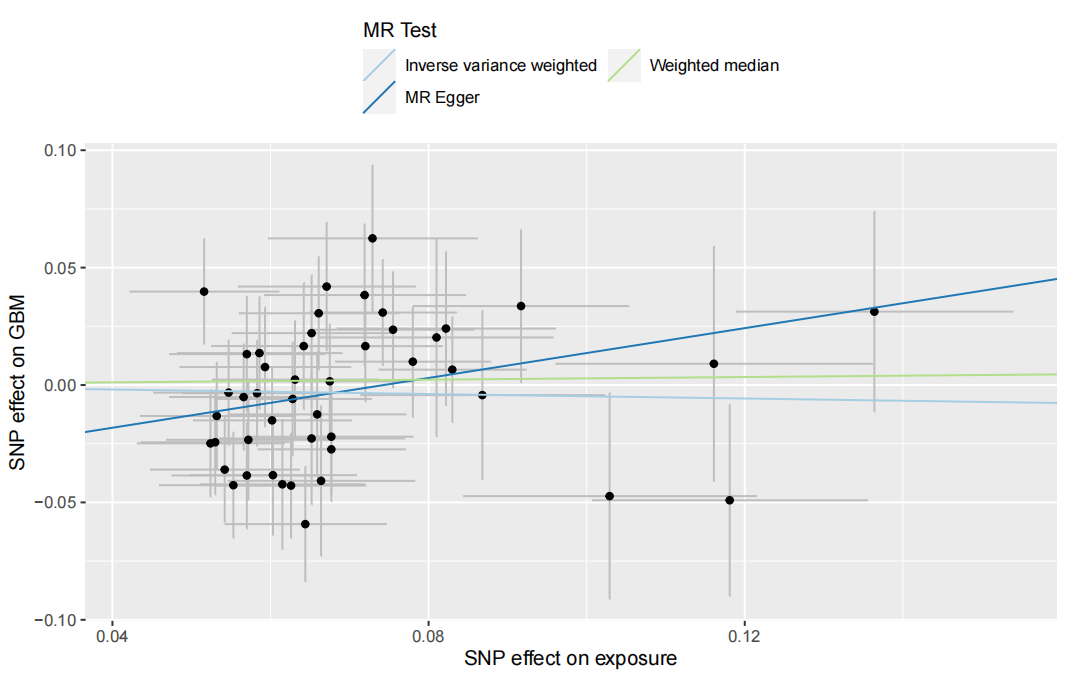


**Supplementary Fig. 12** The leave-one-out plot, funnel plot, and scatter plot for the causal association between bipolar disorder and all-glioma in the primary analysis


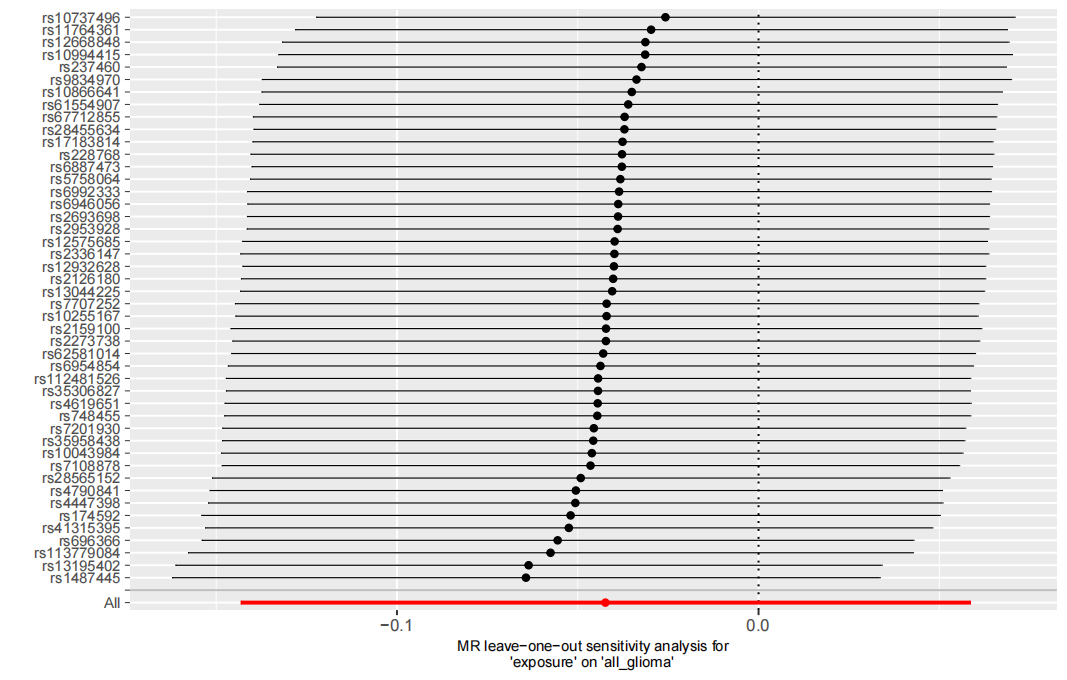


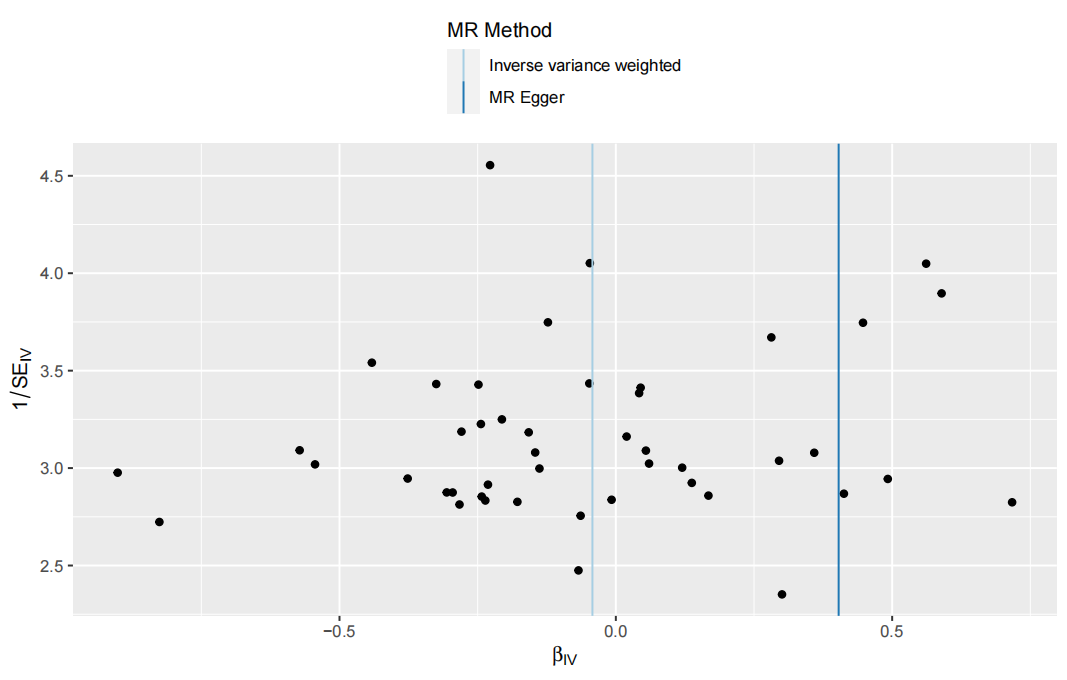


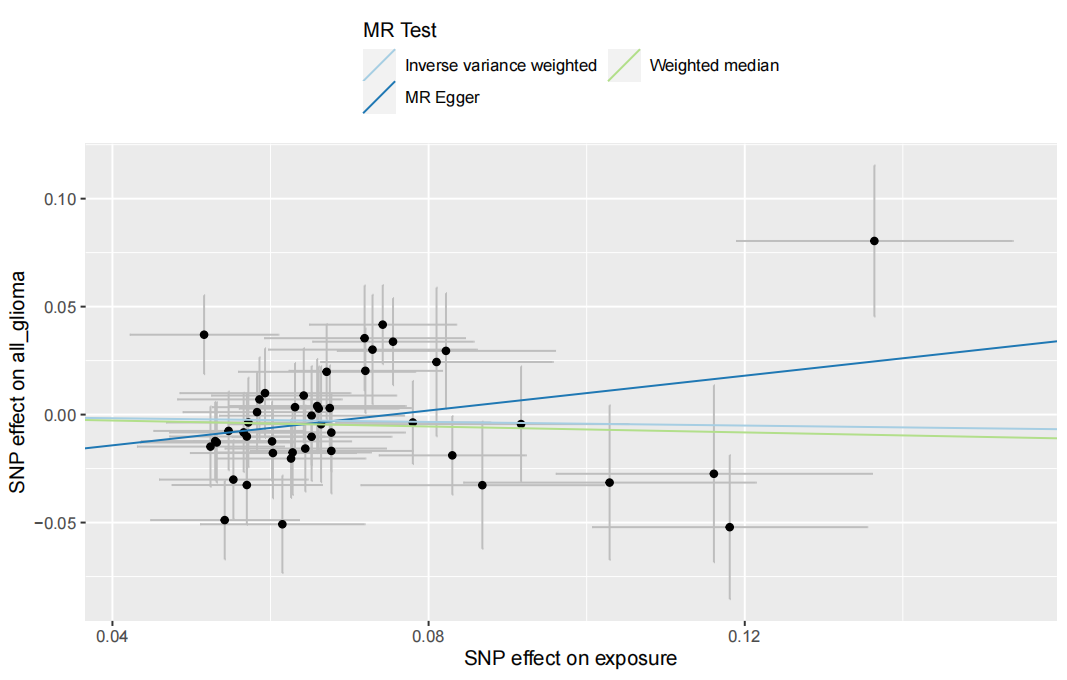


**Supplementary Fig. 13** The leave-one-out plot, funnel plot, and scatter plot for the causal association between major depressive disorder and non-GBM in the primary analysis

**
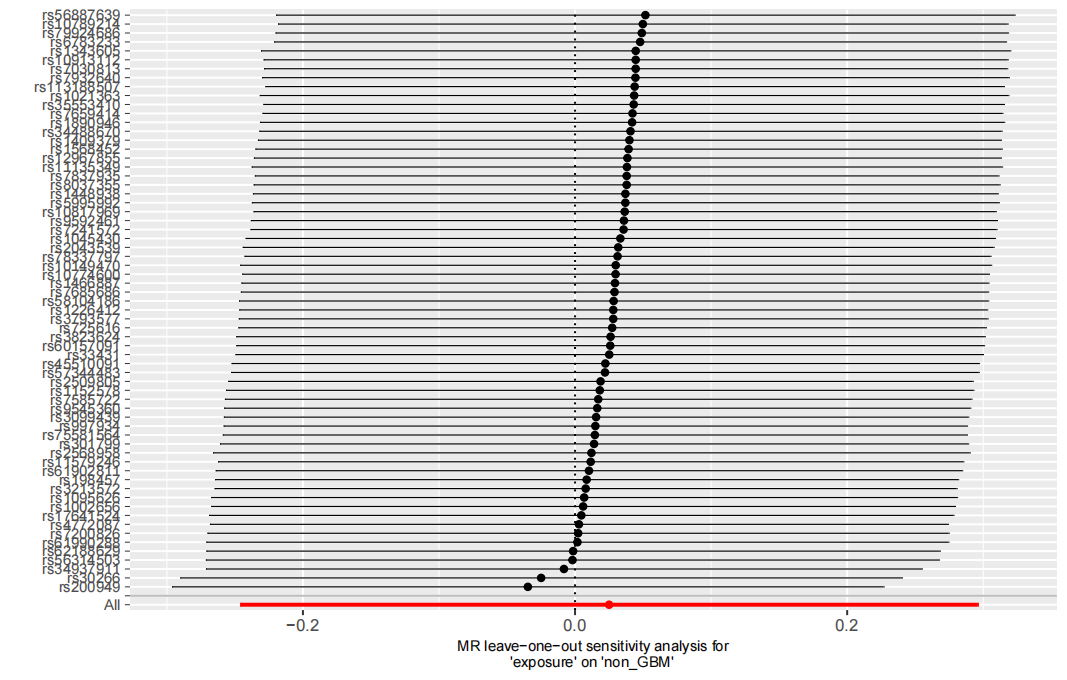
**

**
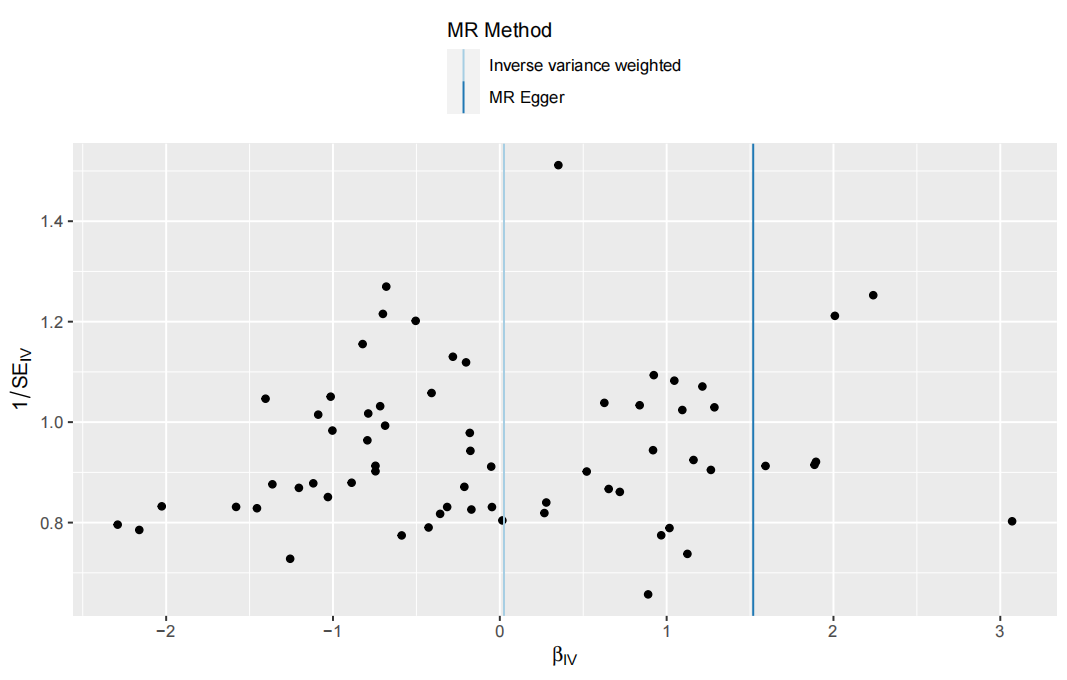
**

**
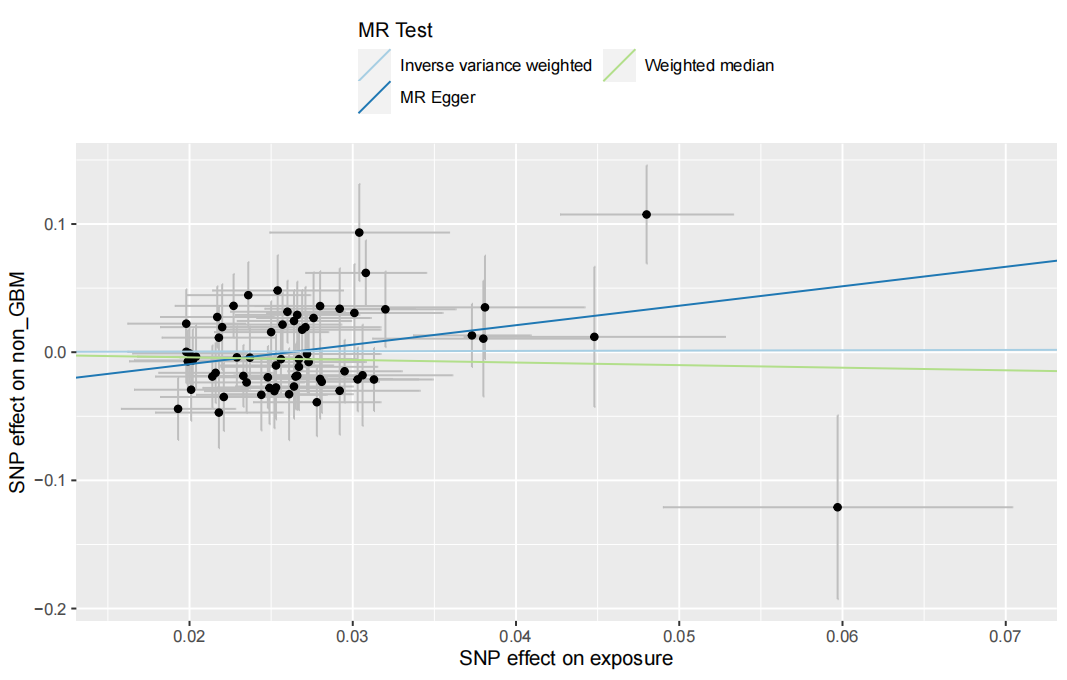
**

**Supplementary Fig. 14** The leave-one-out plot, funnel plot, and scatter plot for the causal association between major depressive disorder and GBM in the primary analysis

**
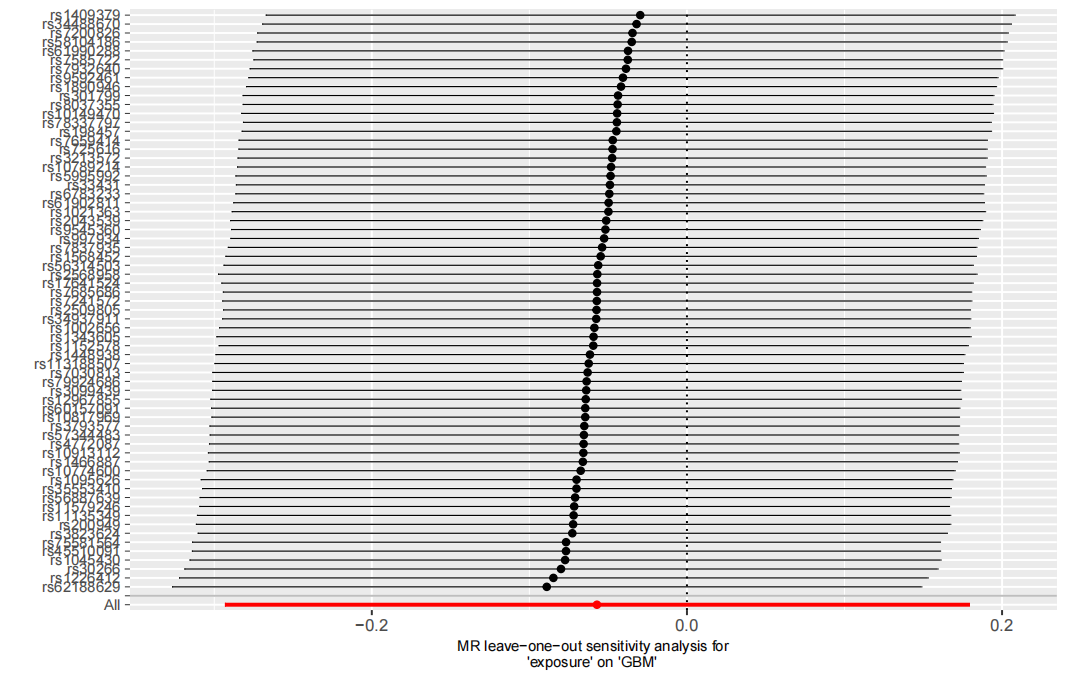
**

**
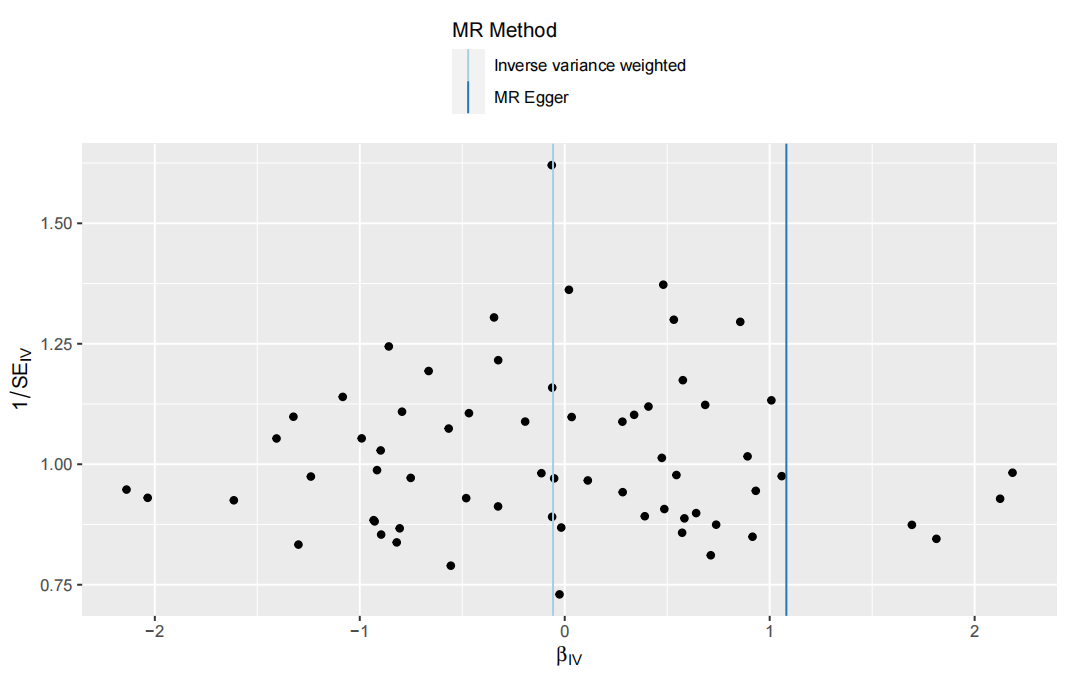
**

**
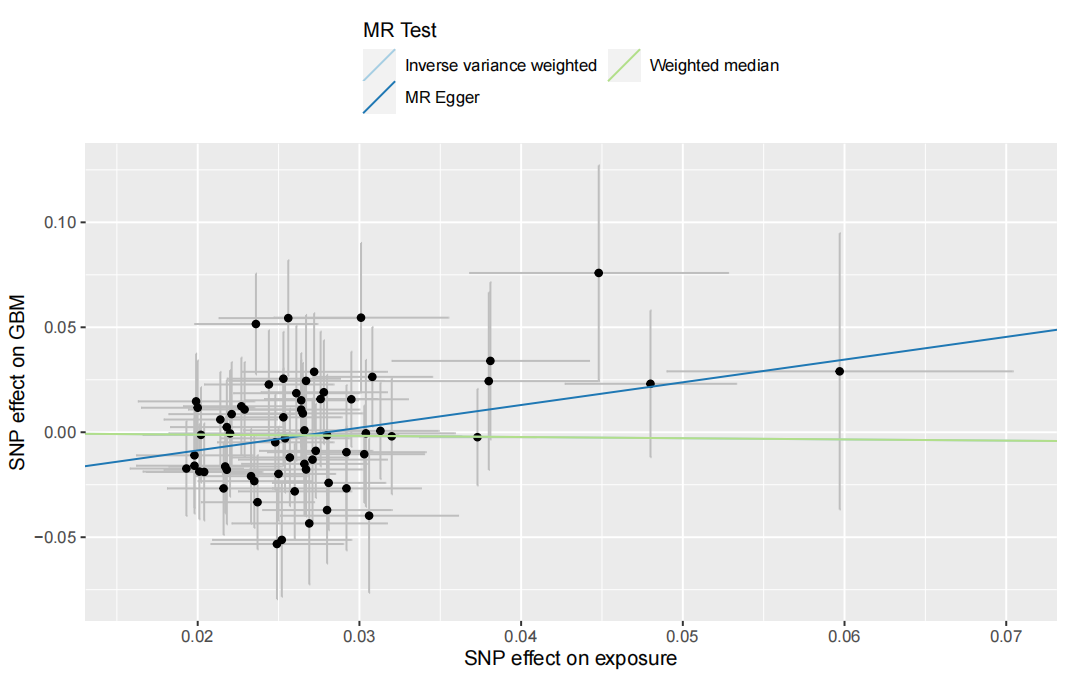
**

**Supplementary Fig. 15** The leave-one-out plot, funnel plot, and scatter plot for the causal association between major depressive disorder and all-glioma in the primary analysis

**
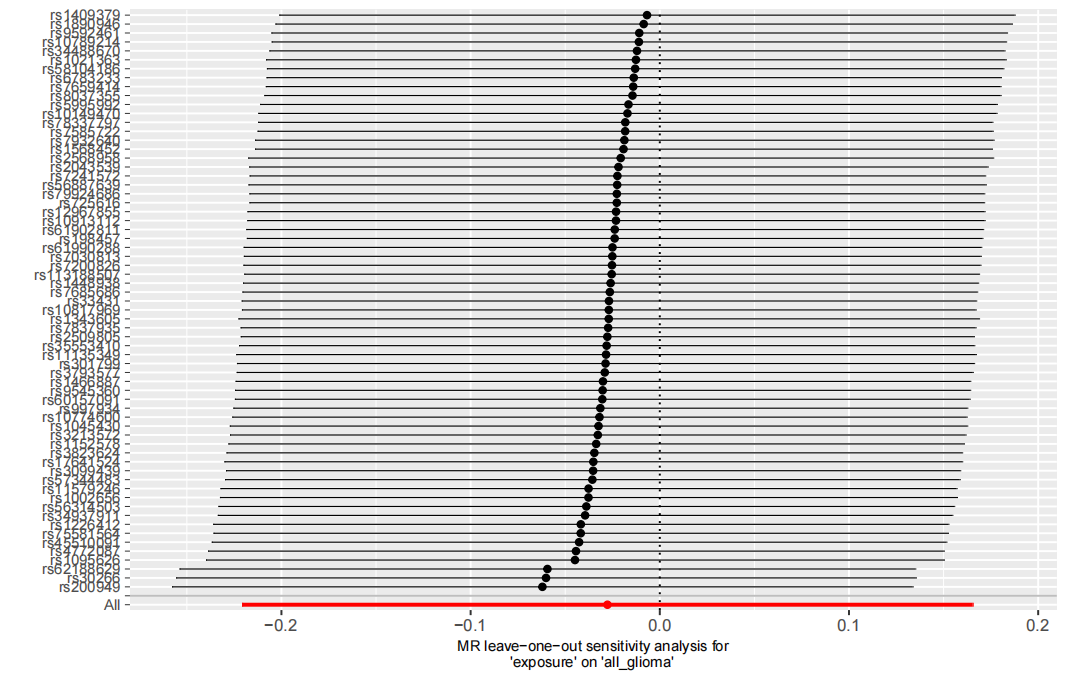
**


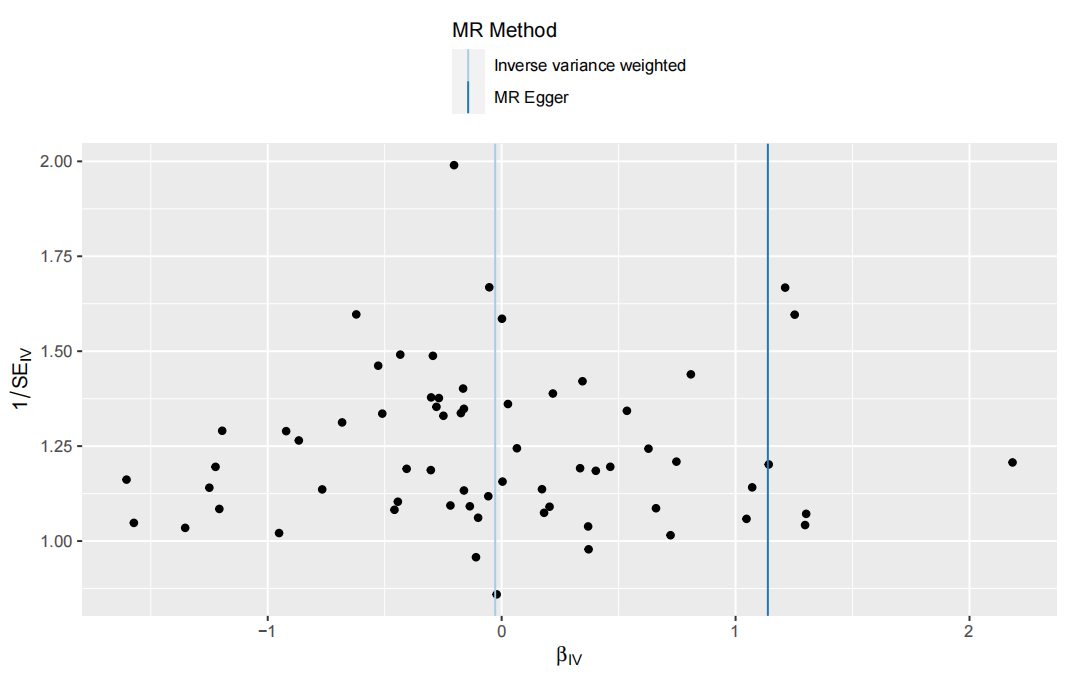


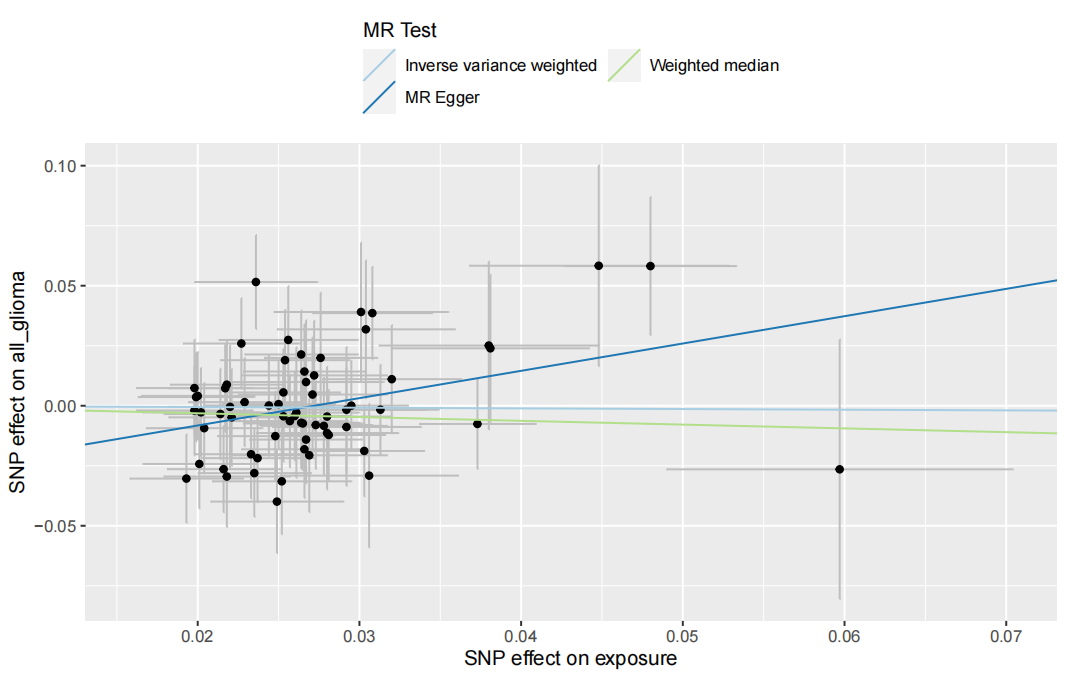


**Supplementary Fig. 16** The leave-one-out plot, funnel plot, and scatter plot for the association between non-GBM and schizophrenia in the reverse MR analysis


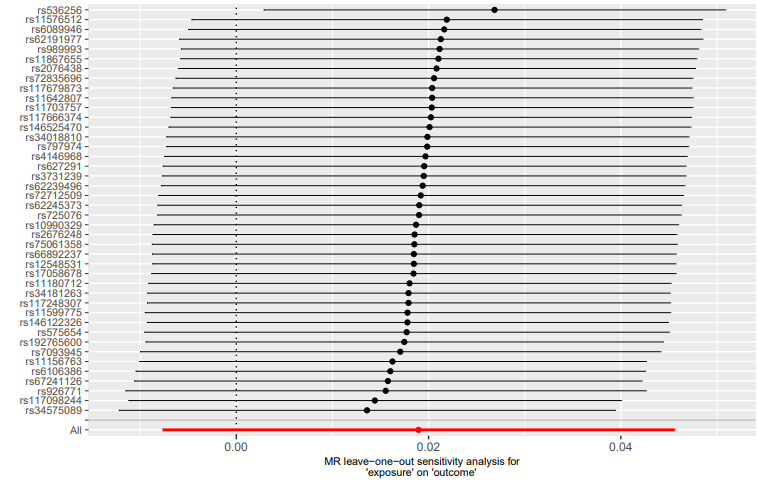


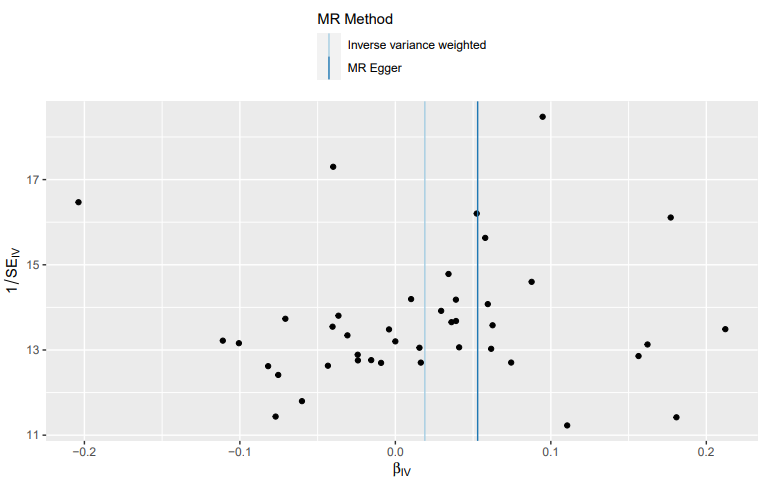


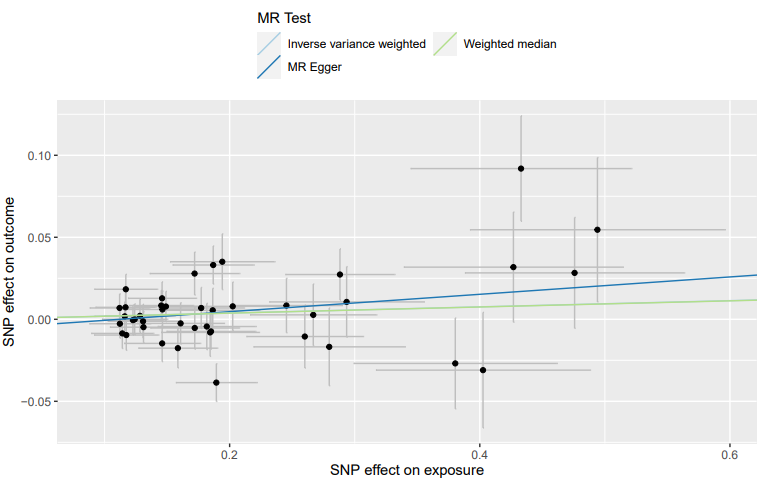


**Supplementary Fig. 17** The leave-one-out plot, funnel plot, and scatter plot for the causal association between GBM and schizophrenia in the reverse MR analysis


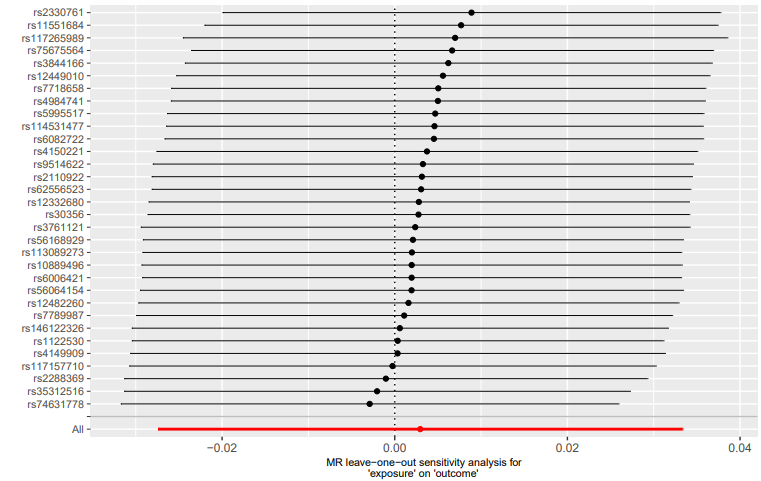


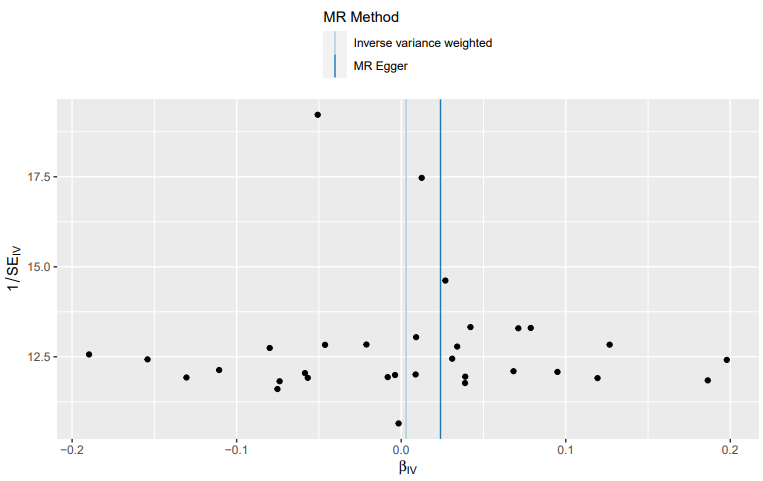


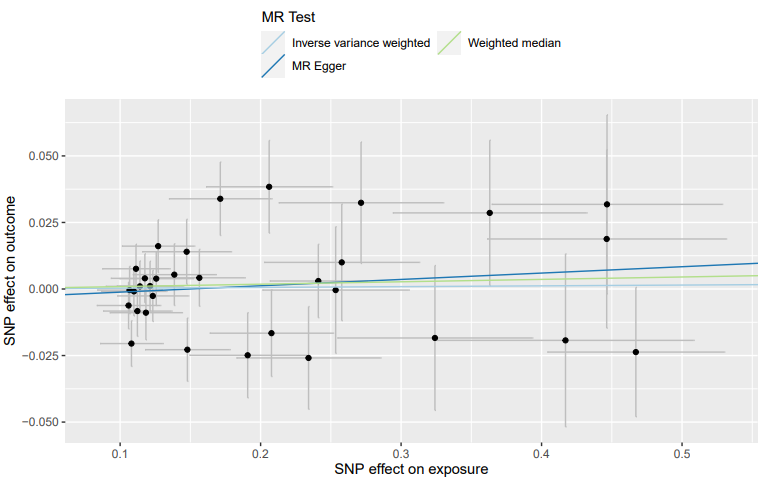


**Supplementary Fig. 18** The leave-one-out plot, funnel plot, and scatter plot for the causal association between all-glioma and schizophrenia in the reverse MR analysis


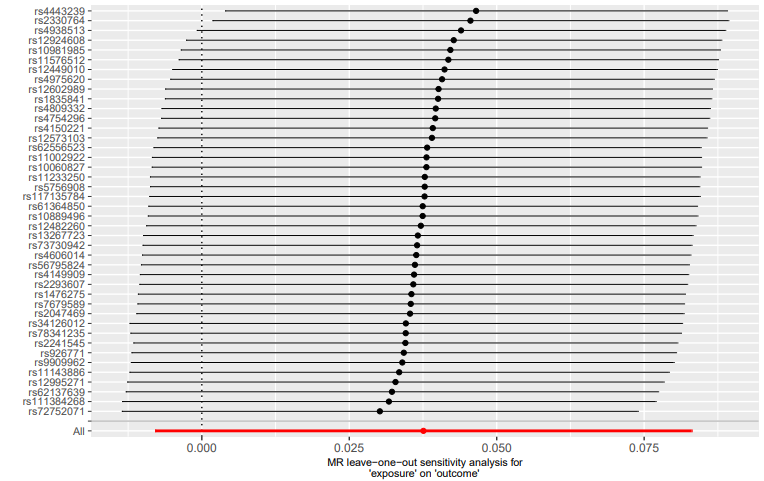


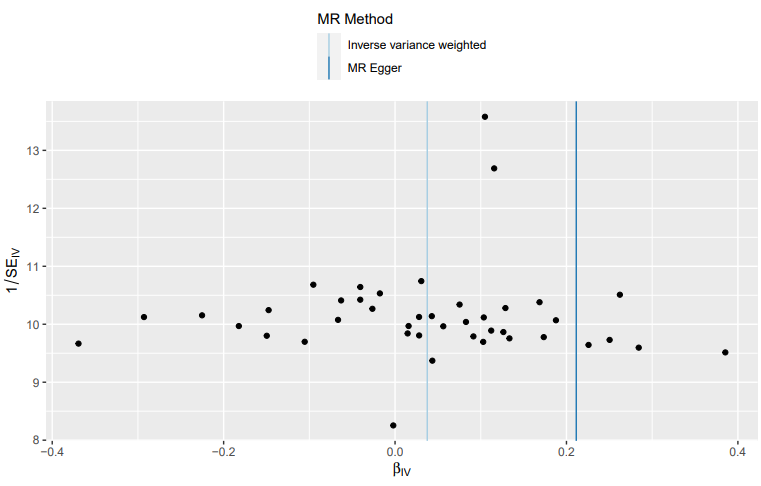


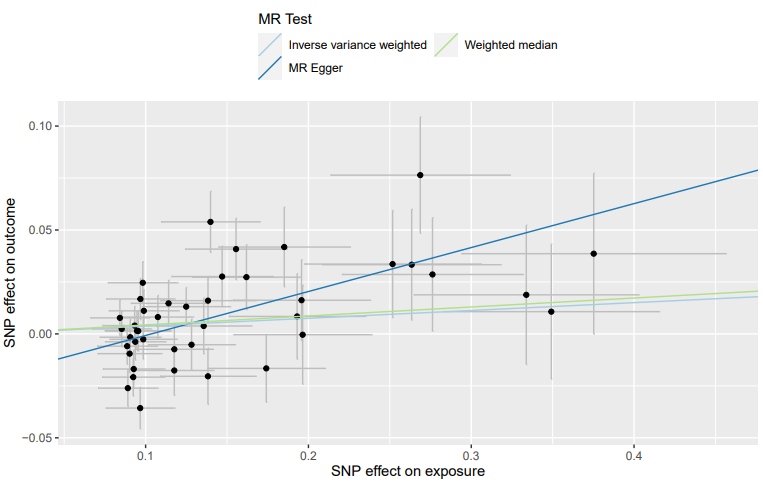


**Supplementary Fig. 19** The leave-one-out plot, funnel plot, and scatter plot for the causal association between non-GBM and panic disorder in the reverse MR analysis


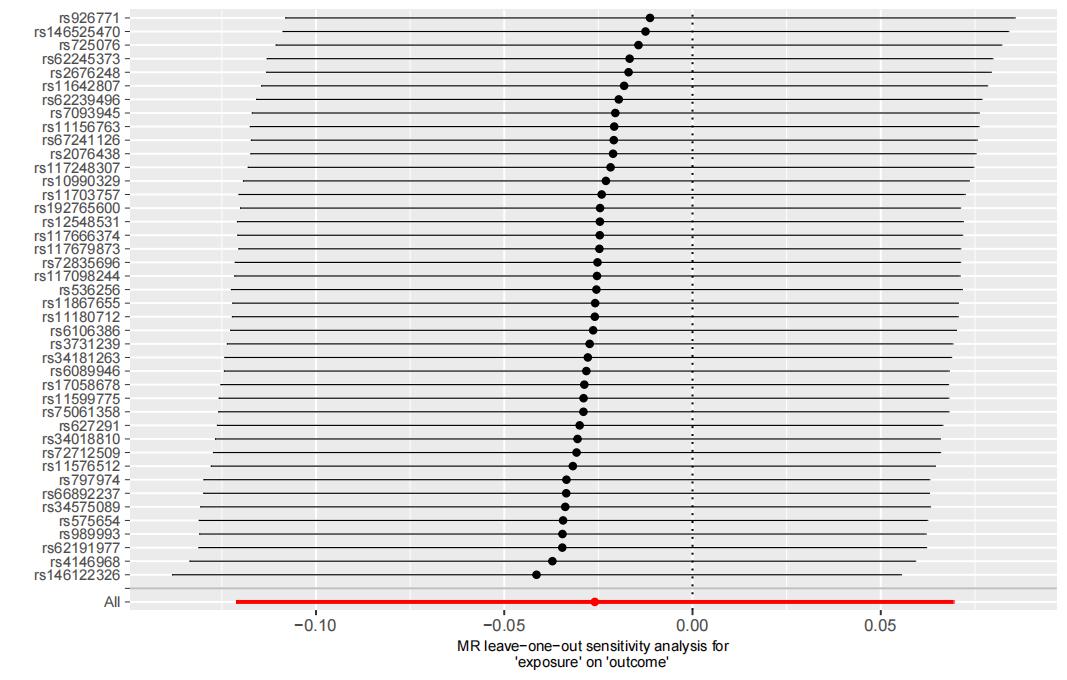


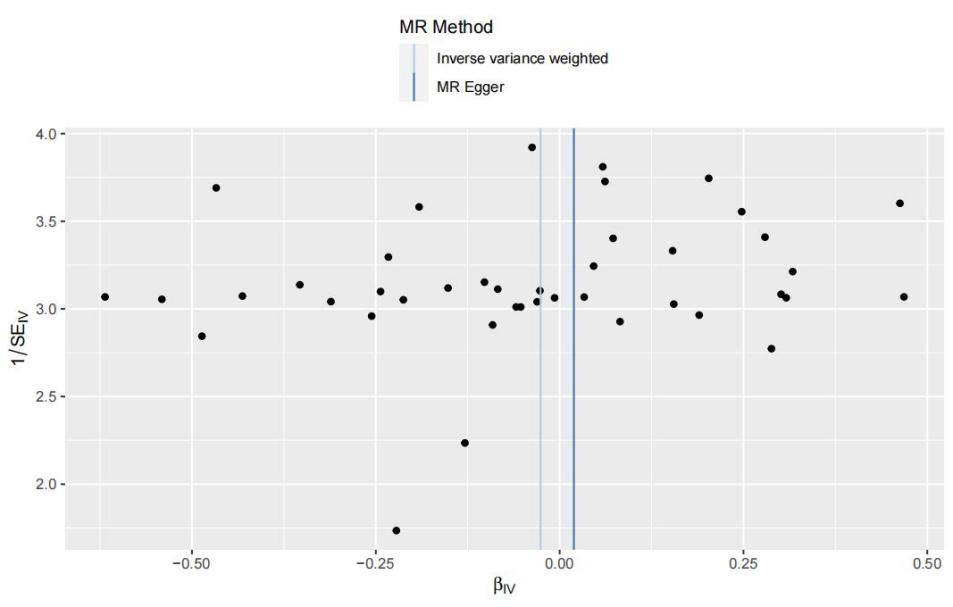


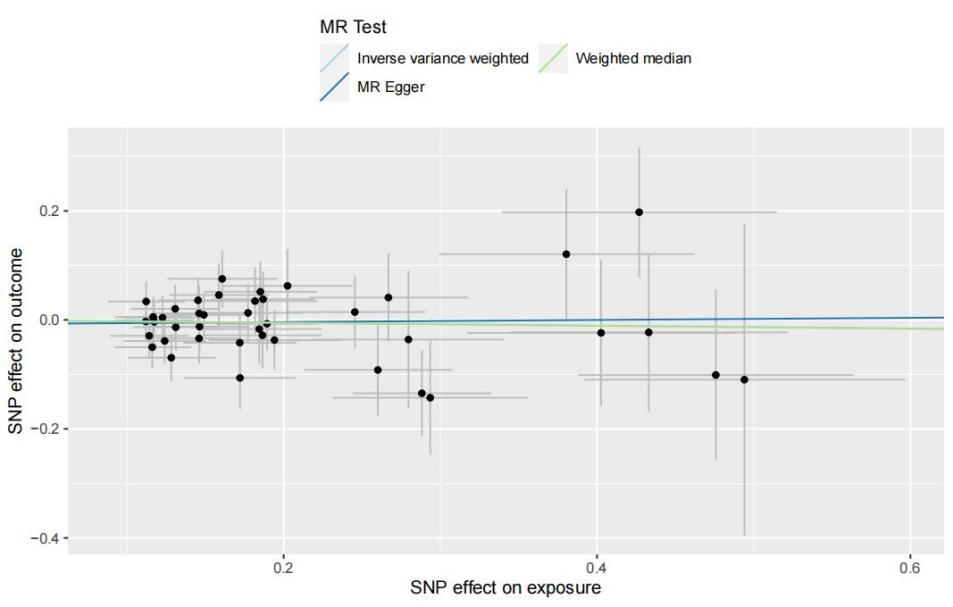


**Supplementary Fig. 20** The leave-one-out plot, funnel plot, and scatter plot for the causal association between GBM and panic disorder in the reverse MR analysis

**
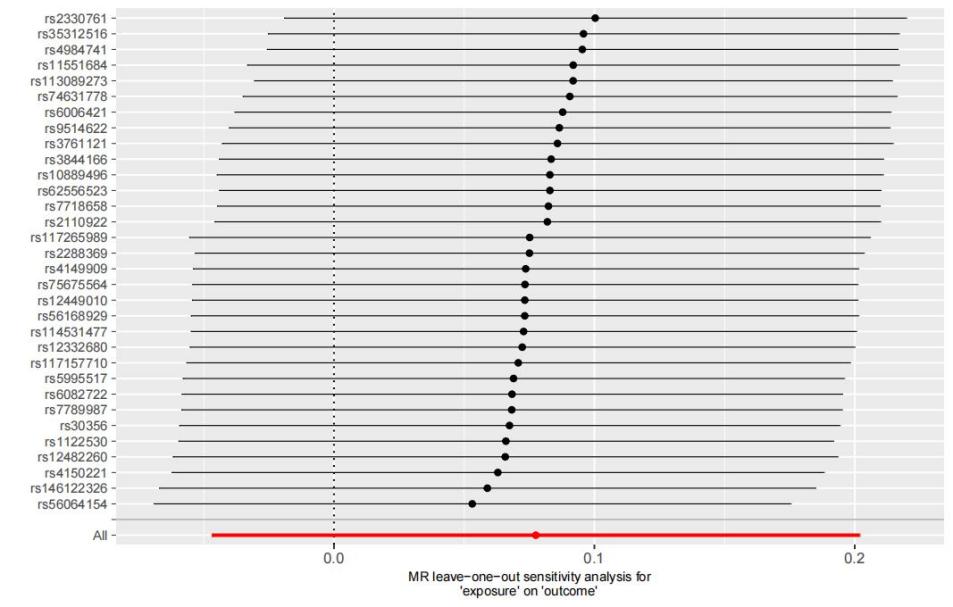
**

**
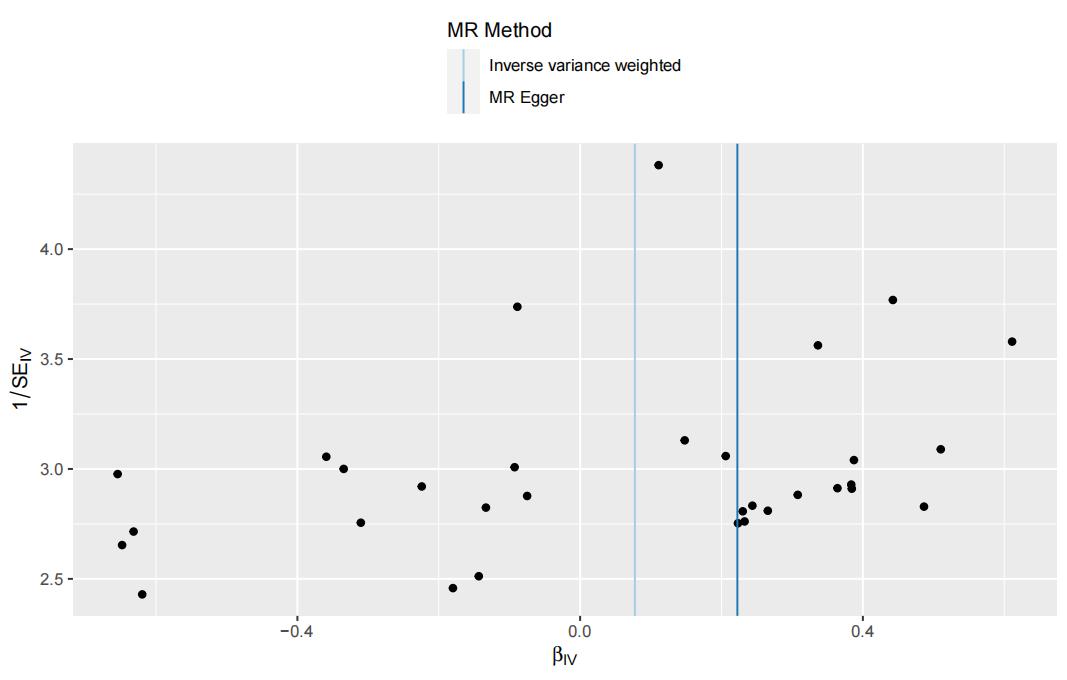
**

**
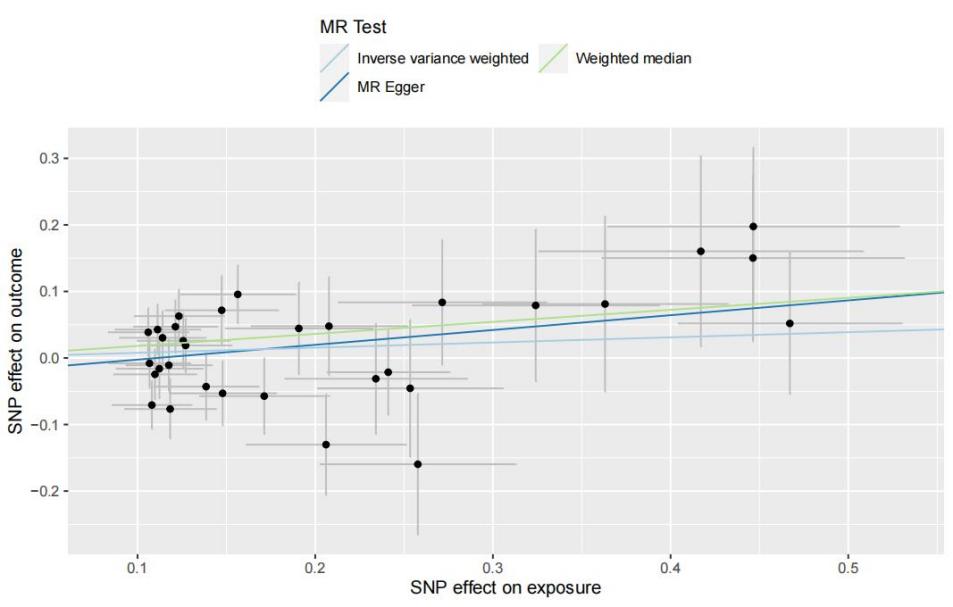
**

**Supplementary Fig. 21** The leave-one-out plot, funnel plot, and scatter plot for the association between all-glioma and panic disorder in the reverse MR analysis

**
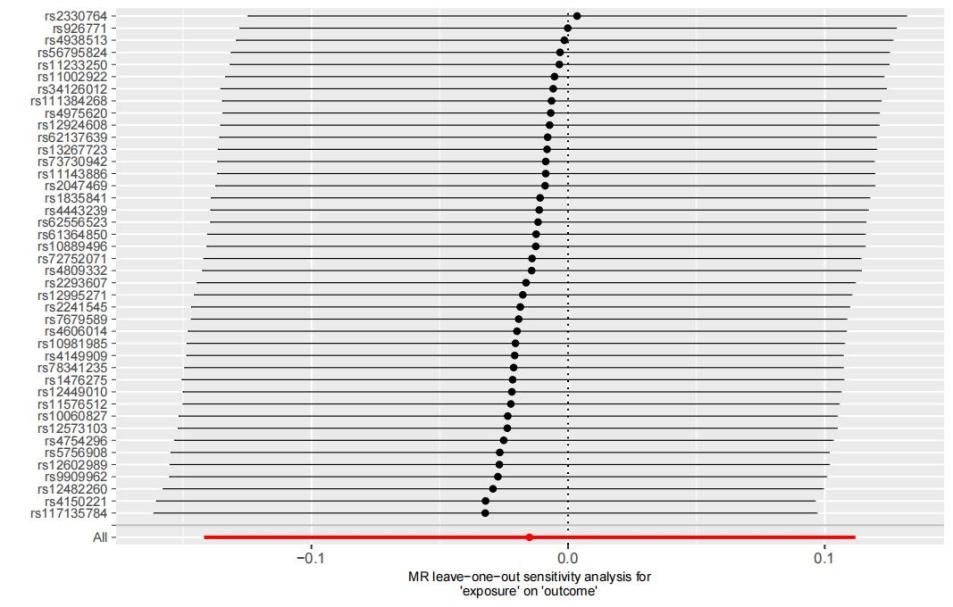
**

**
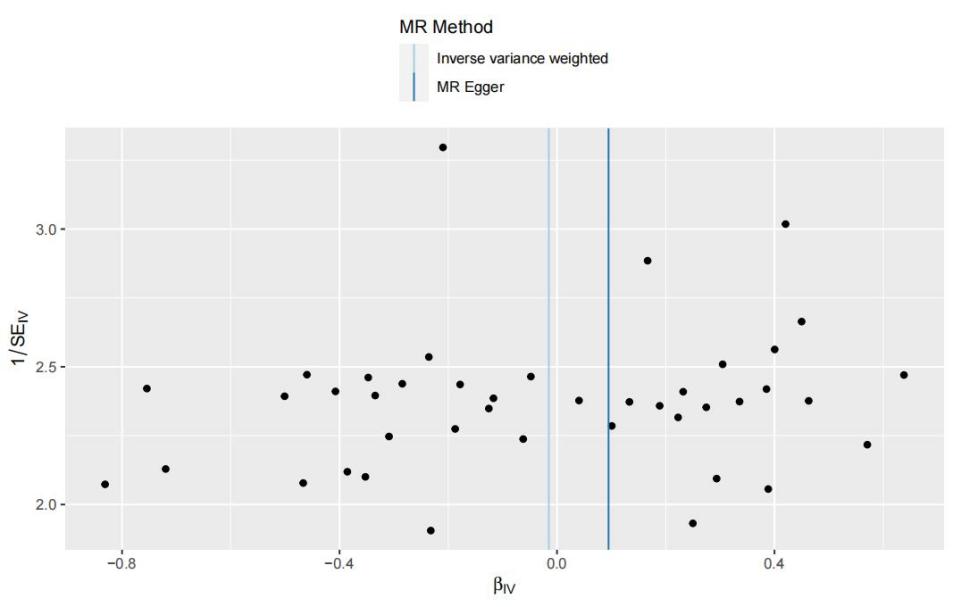
**

**
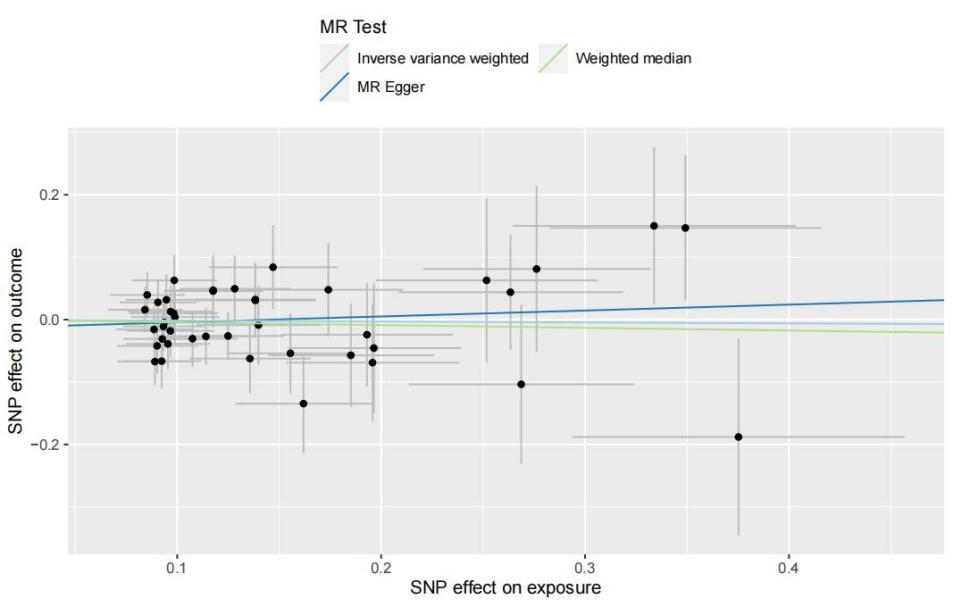
**

**Supplementary Fig. 22** The leave-one-out plot, funnel plot, and scatter plot for the causal association between non-GBM and autistic spectrum disorder in the reverse MR analysis


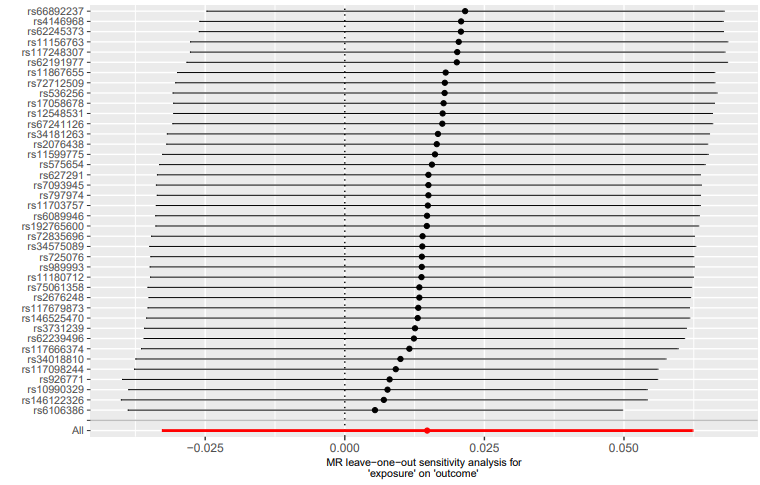


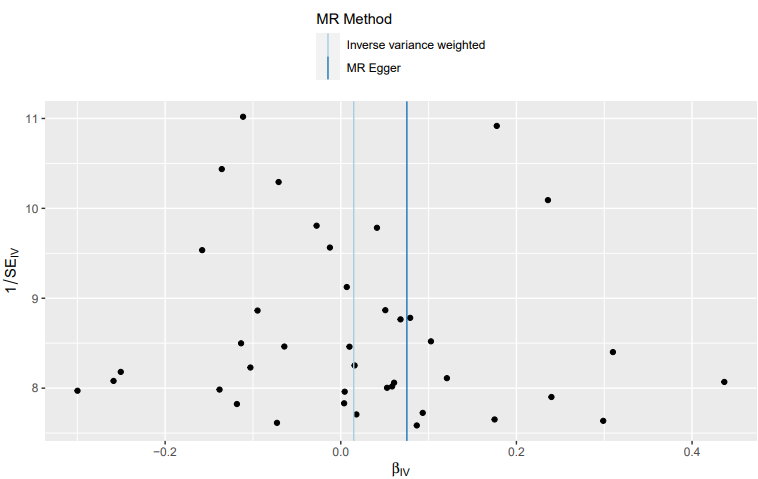


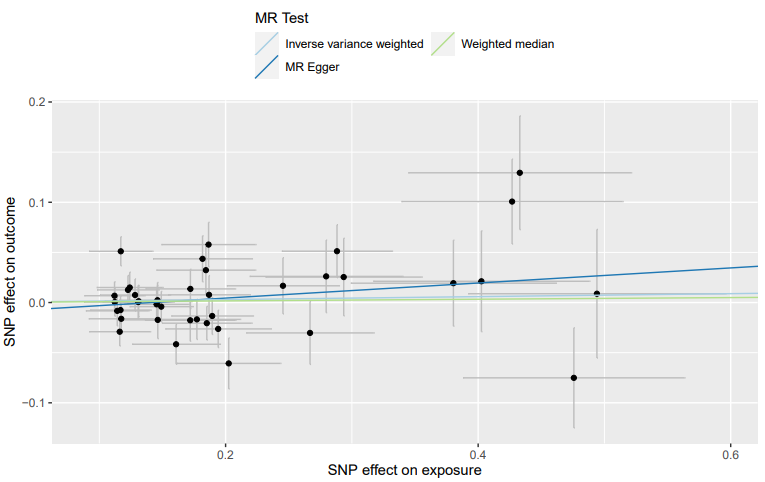


**Supplementary Fig. 23** The leave-one-out plot, funnel plot, and scatter plot for the causal association between GBM and autistic spectrum disorder in the reverse MR analysis


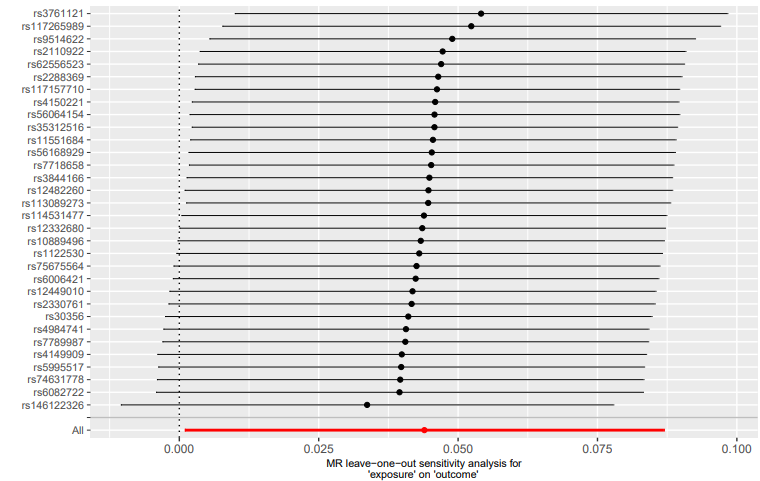


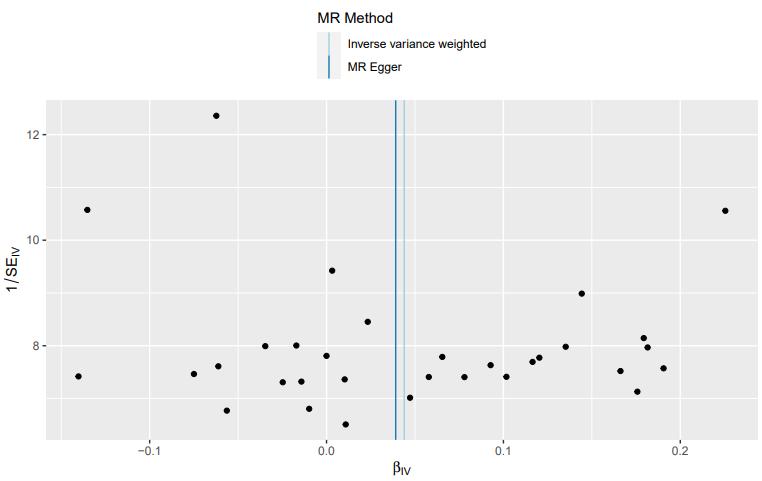


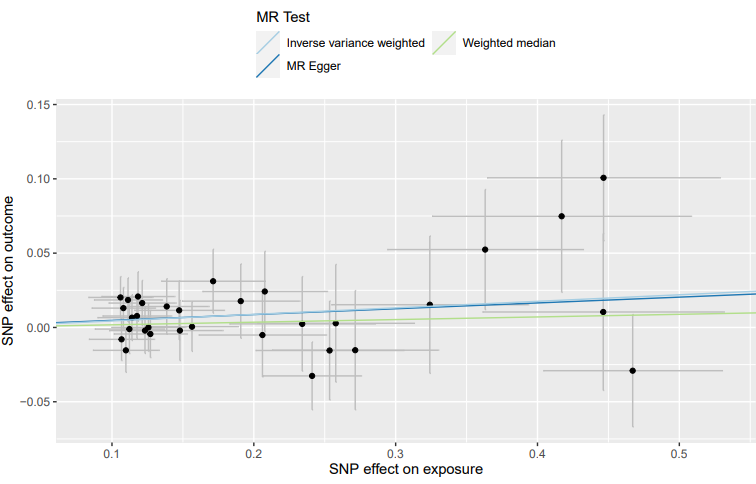


**Supplementary Fig. 24** The leave-one-out plot, funnel plot, and scatter plot for the causal association between all-glioma autistic spectrum disorder in the reverse MR analysis


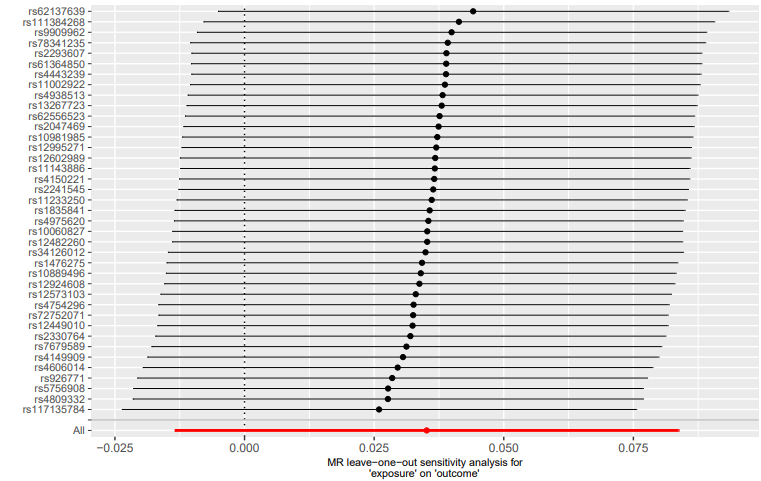


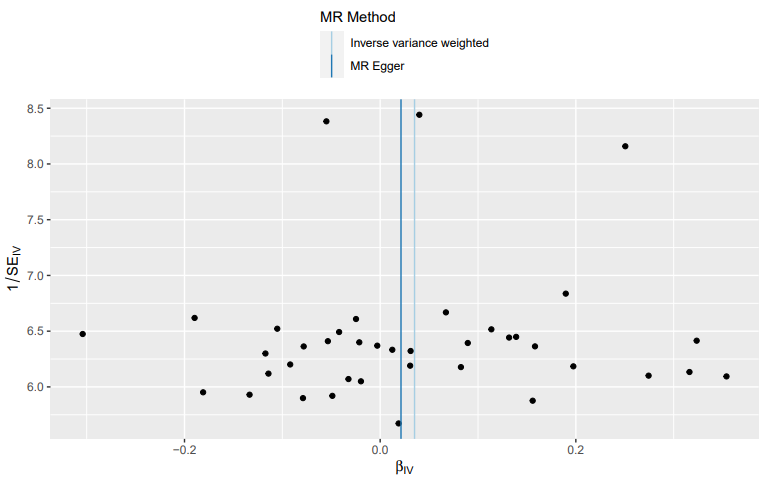


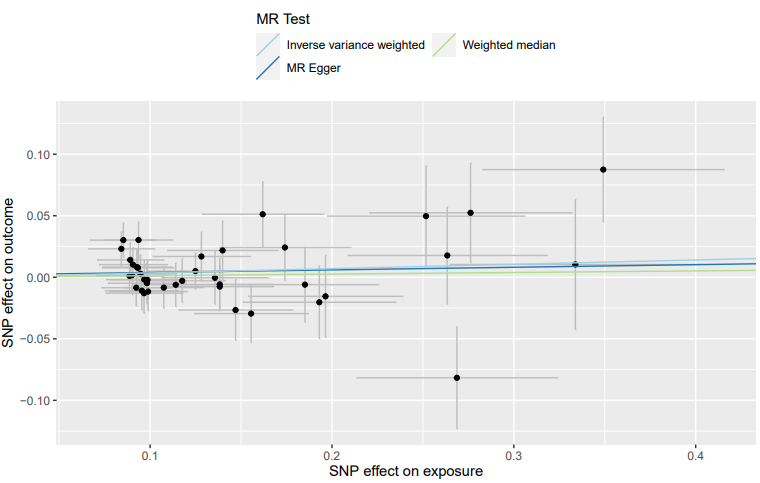


**Supplementary Fig. 25** The leave-one-out plot, funnel plot, and scatter plot for the causal association between non-GBM and bipolar disorder in the reverse MR analysis


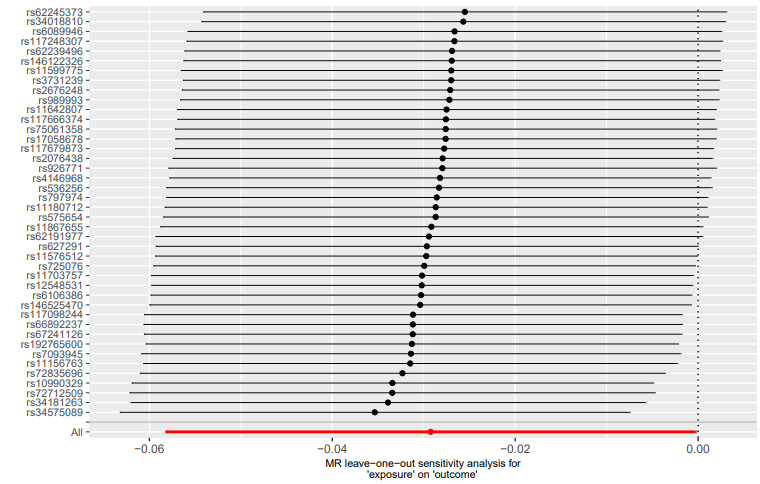


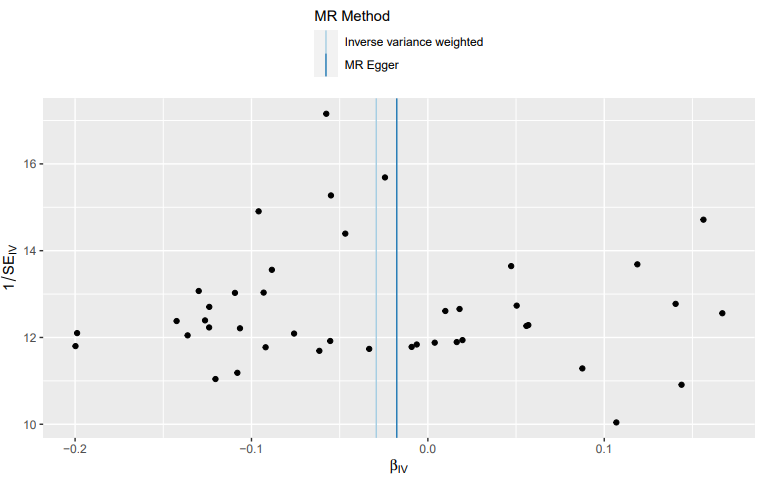


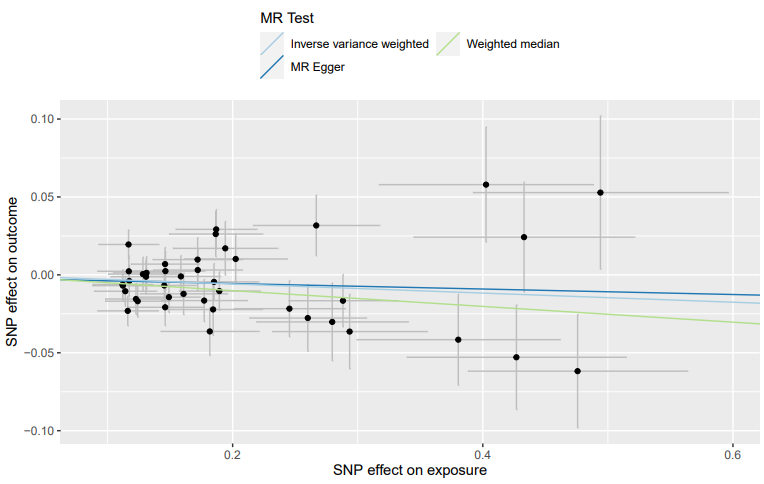


**Supplementary Fig. 26** The leave-one-out plot, funnel plot, and scatter plot for the causal association between GBM and bipolar disorder in the reverse MR analysis


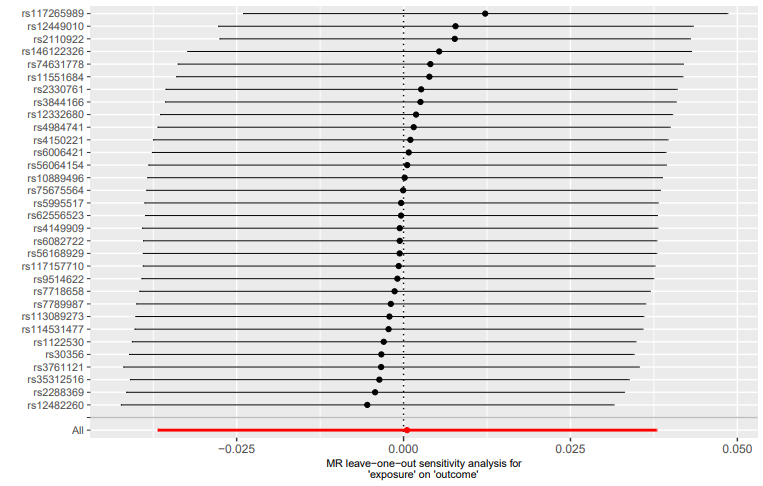


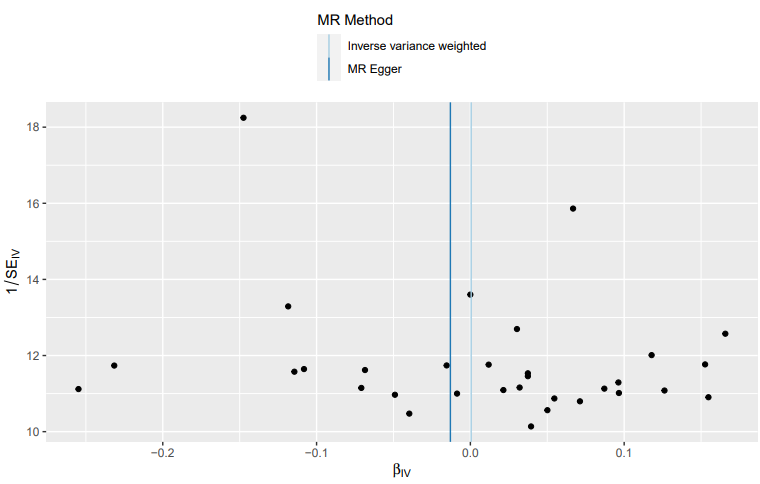


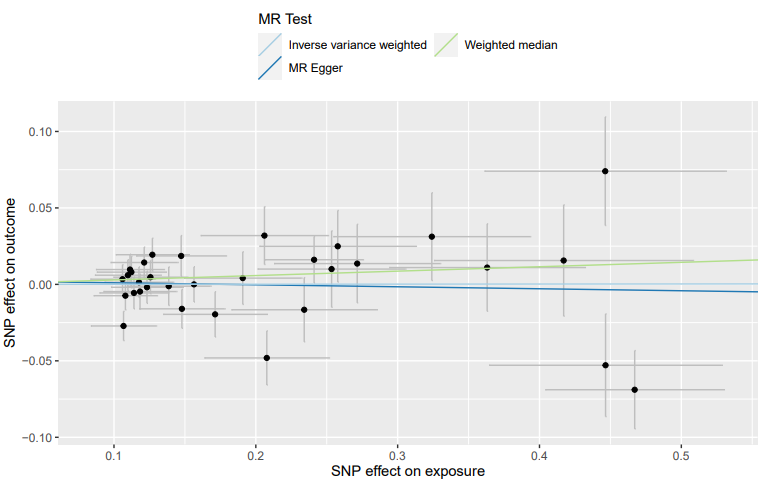


**Supplementary Fig. 27** The leave-one-out plot, funnel plot, and scatter plot for the causal association between all-glioma and bipolar disorder in the reverse MR analysis


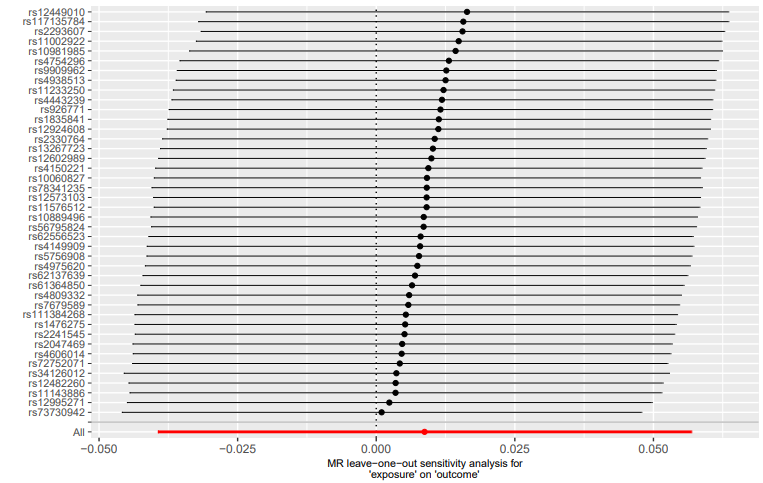


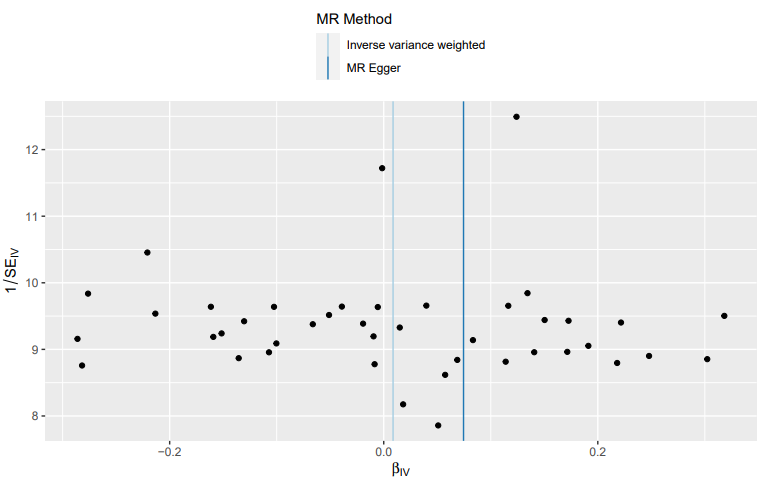


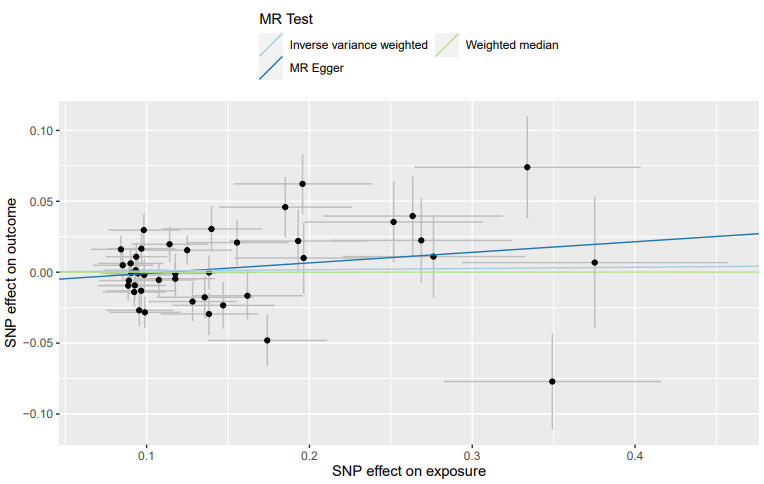


**Supplementary Fig. 28** The leave-one-out plot, funnel plot, and scatter plot for the causal association between non-GBM and major depressive disorder in the reverse MR analysis


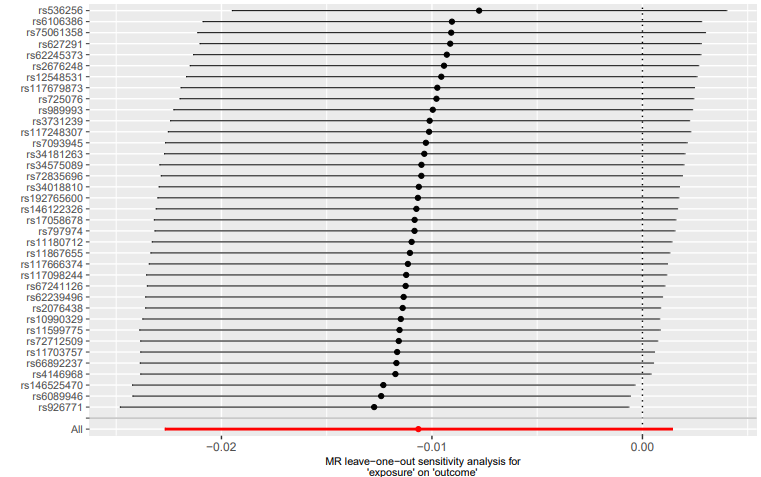


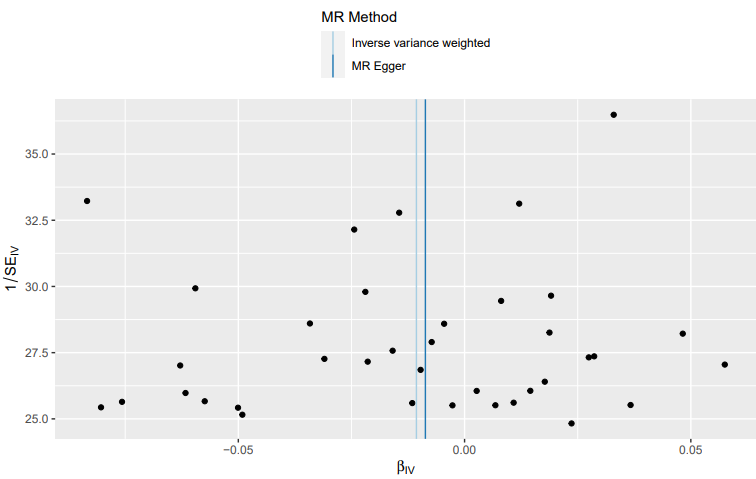


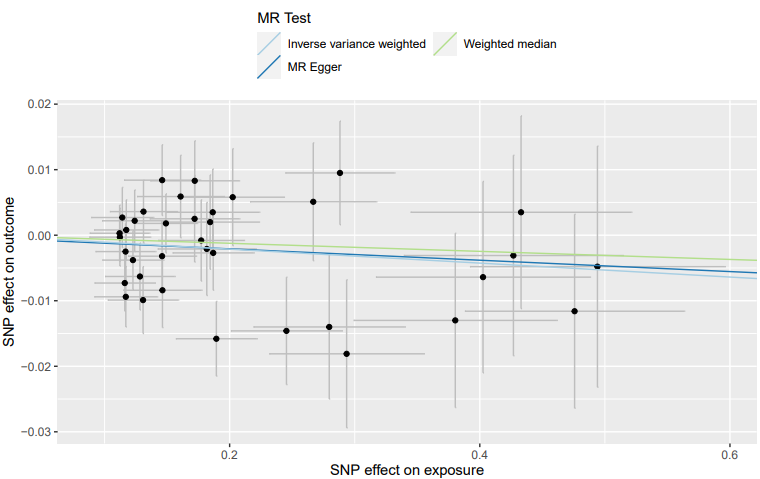


**Supplementary Fig. 29** The leave-one-out plot, funnel plot, and scatter plot for the causal association between GBM and major depressive disorder in the reverse MR analysis


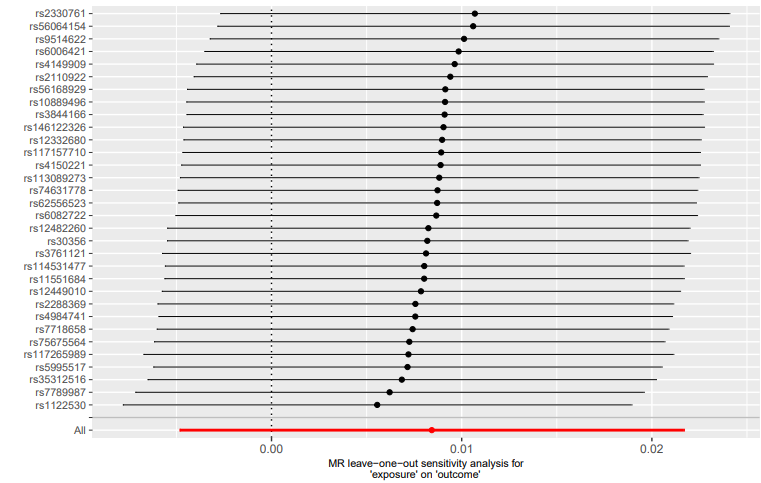


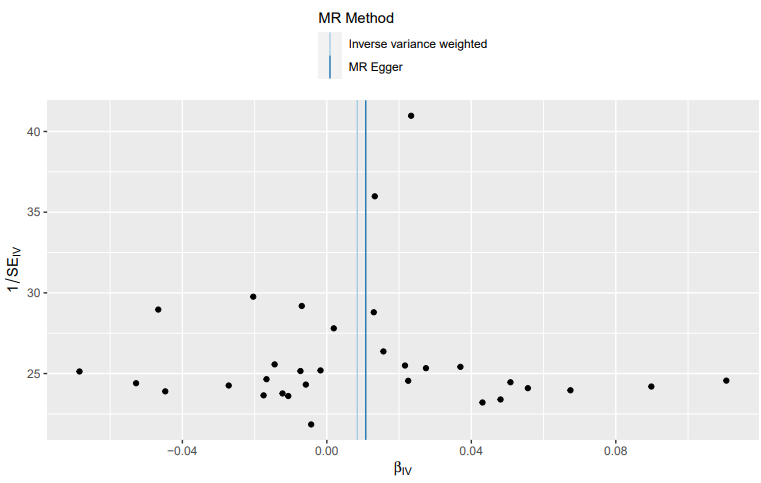


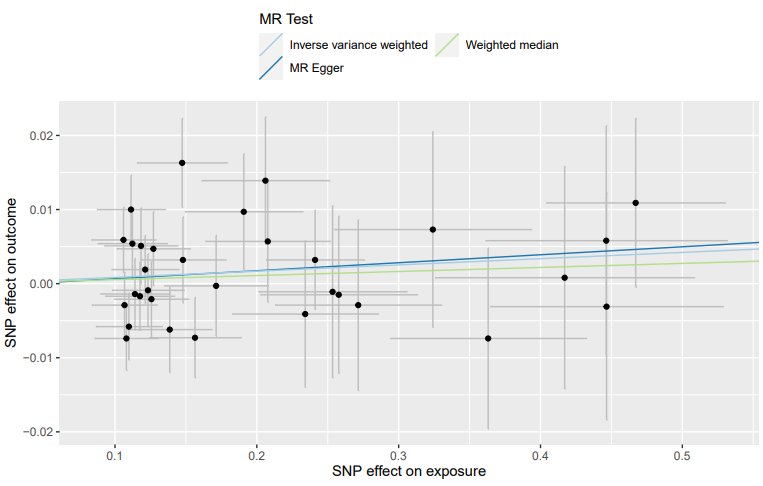


**Supplementary Fig. 30** The leave-one-out plot, funnel plot, and scatter plot for the association of all-glioma and major depressive disorder in the reverse MR analysis


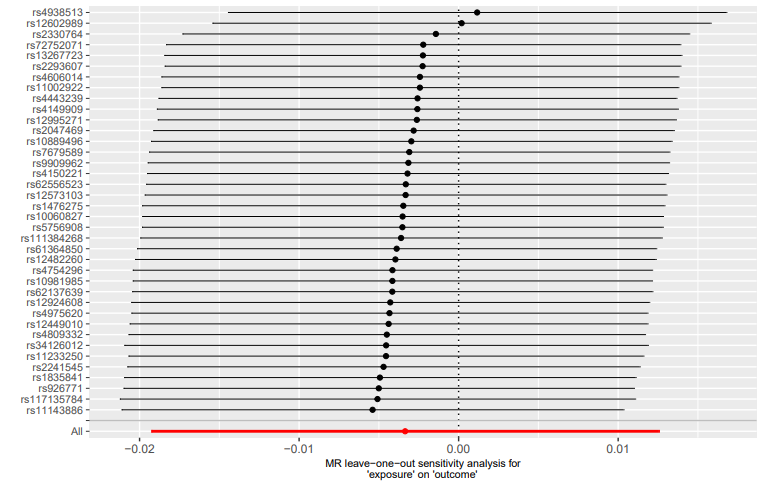


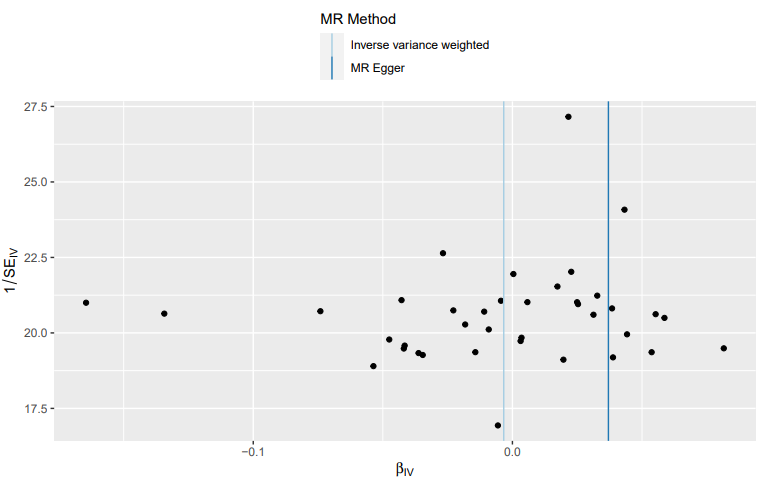


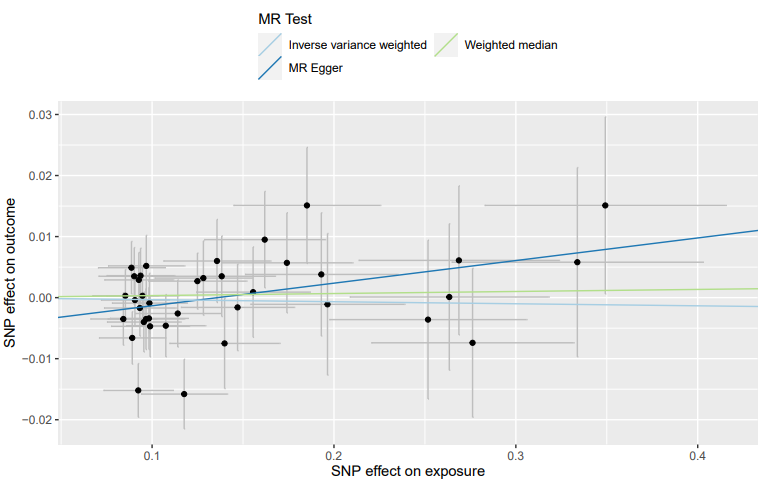

Supplement: Supplementary file 3 — Supplementary Fig. 1 The leave-one-out plot, funnel plot, and scatter plot for the causal association between schizophrenia and non-GBM in the primary analysis. Supplementary Fig. 2 The leave-one-out plot, funnel plot, and scatter plot for the causal association between of schizophrenia and GBM in the primary analysis. Supplementary Fig. 3 The leave-one-out plot, funnel plot, and scatter plot for the causal association between schizophrenia and all-glioma in the primary analysis. Supplementary Fig. 4 The leave-one-out plot, funnel plot, and scatter plot for the causal association between panic disorder and non-GBM in the primary analysis. Supplementary Fig. 5 The leave-one-out plot, funnel plot, and scatter plot for the causal association between panic disorder and GBM in the primary analysis. Supplementary Fig. 6 The leave-one-out plot, funnel plot, and scatter plot for the causal association between panic disorder and all-glioma in the primary analysis. Supplementary Fig. 7 The leave-one-out plot, funnel plot, and scatter plot for the causal association between autistic spectrum disorder and non-GBM in the primary analysis. Supplementary Fig. 8 The leave-one-out plot, funnel plot, and scatter plot for the causal association between autistic spectrum disorder and GBM in the primary analysis. Supplementary Fig. 9 The leave-one-out plot, funnel plot, and scatter plot for the causal association between autistic spectrum disorder and all-glioma in the primary analysis. Supplementary Fig. 10 The leave-one-out plot, funnel plot, and scatter plot for the causal association between bipolar disorder and non-GBM in the primary analysis. Supplementary Fig. 11 The leave-one-out plot, funnel plot, and scatter plot for the causal association between bipolar disorder and GBM in the primary analysis. Supplementary Fig. 12 The leave-one-out plot, funnel plot, and scatter plot for the causal association between bipolar disorder and all-glioma in the primary analysis. Supplementary F [file 12885_2024_11865_MOESM3_ESM.docx]
